# Analysis of the risk and pre-emptive control of viral outbreaks accounting for within-host dynamics: SARS-CoV-2 as a case study

**DOI:** 10.1073/pnas.2305451120

**Published:** 2023-10-03

**Authors:** William S. Hart, Hyeongki Park, Yong Dam Jeong, Kwang Su Kim, Raiki Yoshimura, Robin N. Thompson, Shingo Iwami

**Affiliations:** ^a^Mathematical Institute, University of Oxford, Oxford OX2 6GG, United Kingdom; ^b^lnterdisciplinary Biology Laboratory, Division of Natural Science, Graduate School of Science, Nagoya University, Nagoya 464-8602, Japan; ^c^Department of Mathematics, Pusan National University, Busan 46241, South Korea; ^d^Department of Scientific Computing, Pukyong National University, Busan 48513, South Korea; ^e^Mathematics Institute, University of Warwick, Coventry CV4 7AL, United Kingdom; ^f^Zeeman Institute for Systems Biology and Infectious Disease Epidemiology Research, University of Warwick, Coventry CV4 7AL, United Kingdom; ^g^Institute of Mathematics for Industry, Kyushu University, Fukuoka 819-0395, Japan; ^h^Institute for the Advanced Study of Human Biology, Kyoto University, Kyoto 606-8501, Japan; ^i^Interdisciplinary Theoretical and Mathematical Sciences Program, RIKEN, Saitama 351-0198, Japan; ^j^NEXT-Ganken Program, Japanese Foundation for Cancer Research, Tokyo 135-8550, Japan; ^k^Science Groove Inc., Fukuoka 810-0041, Japan

**Keywords:** infectious disease modelling, outbreak risk, SARS-CoV-2, COVID-19, antigen testing

## Abstract

In the era of living with COVID-19, the risk of localised SARS-CoV-2 outbreaks remains. Here, we develop a multiscale modelling framework for estimating the local outbreak risk for a viral disease (the probability that a major outbreak results from a single case introduced into the population), accounting for within-host viral dynamics. Compared to population-level models previously used to estimate outbreak risks, our approach enables more detailed analysis of how the risk can be mitigated through pre-emptive interventions such as antigen testing. Considering SARS-CoV-2 as a case study, we quantify the within-host dynamics using data from individuals with omicron variant infections. We demonstrate that regular antigen testing reduces, but may not eliminate, the outbreak risk, depending on characteristics of local transmission. In our baseline analysis, daily antigen testing reduces the outbreak risk by 45% compared to a scenario without antigen testing. Additionally, we show that accounting for heterogeneity in within-host dynamics between individuals affects outbreak risk estimates and assessments of the impact of antigen testing. Our results therefore highlight important factors to consider when using multiscale models to design pre-emptive interventions against SARS-CoV-2 and other viruses.

## Significance

Estimates of the risk of viral disease outbreaks occurring in different populations are important for effective allocation of limited surveillance and control resources. Here, we show how changes in viral load during infection can be included in outbreak risk calculations and demonstrate how intervention effectiveness can be assessed in greater detail by considering within-host viral dynamics. We focus on the risk of localised SARS-CoV-2 outbreaks due to the omicron variant. We find that regular population-wide antigen testing is likely to reduce the outbreak risk, but not prevent outbreaks entirely, depending on characteristics of the local population. Our results highlight the importance of considering factors such as heterogeneity in within-host viral dynamics for outbreak risk estimation and analysis of interventions.

Following the widespread rollout of COVID-19 vaccines, countries worldwide have adopted “living with COVID-19” policies [for example, the United Kingdom removed its final domestic restrictions in February 2022 (1)]. Waves of COVID-19 cases continue to occur (2, 3), generated by factors including waning immunity (4–8) and the continued evolution of the severe acute respiratory syndrome coronavirus 2 (SARS-CoV-2) virus (3, 6, 9–13), although vaccines provide high levels of ongoing protection against severe disease (8). Nonetheless, localised outbreaks, either in geographical areas or in specific populations such as schools, universities and workplaces, continue to cause disruption (for example, through student or staff absence).

Mathematical modelling can be used to estimate the (local) *outbreak risk*, which is defined as the probability that a major infectious disease outbreak results from a single infection introduced to the population (14–18). While the outbreak risk can be estimated by simulating a stochastic epidemic model a large number of times (and calculating the proportion of simulations in which a major outbreak occurs), branching process theory can also be used to derive outbreak risk estimates analytically (18). A commonly used analytic outbreak risk estimate in the applied epidemic modelling literature (19–25) is $1-1/R_{0}$   (whenever the basic reproduction number, $R_{0}>1$   ; when $R_{0}\leq 1$   , the outbreak risk is zero). However, this formula relies on simplistic assumptions, including each infected individual having constant infectiousness throughout an exponentially distributed infectious period. Several studies have therefore relaxed these assumptions, for example, by considering a gamma-distributed infectious period (20, 23, 26) and/or accounting for heterogeneity between age groups (15, 27, 28).

In multiscale epidemic modelling frameworks, within-host viral dynamics models, which describe how the viral load of an infected host evolves over the course of infection and can be calibrated using longitudinal individual data, are used to inform population-level epidemiological models (29–31). One advantage of such approaches is that they facilitate a detailed description of the impact of interventions, such as antigen testing (32–34) or the use of antiviral drugs (35), which depend upon and/or affect within-host dynamics in a manner that cannot be fully captured in simple population-level models. Multiscale modelling approaches have been applied to SARS-CoV-2 (32–39) and other pathogens including influenza (29, 40) to generate outbreak projections and test control interventions. However, to our knowledge, multiscale methods have not previously been used to estimate the outbreak risk, or to analyse how this risk can be mitigated through pre-emptive interventions.

In this study, we develop a multiscale modelling approach for calculating the outbreak risk, accounting for within-host viral dynamics and heterogeneity in these dynamics between individuals. We derive an equation satisfied by the outbreak risk under a multiscale model and verify our analytically derived outbreak risk estimates using simulations of an individual-based stochastic epidemic model. Focusing on the case study of SARS-CoV-2, we characterise the viral dynamics by fitting a within-host model (41–48) to data from 521 individuals with infections due to the omicron variant (49). We first consider the outbreak risk in the absence of interventions, before exploring the extent to which the outbreak risk can be mitigated through regular rapid antigen testing of the entire local population. Additionally, we analyse the impact of the reproduction number for local transmissions, the level of transmission following detection, heterogeneity in within-host dynamics, and asymptomatic infection, on the outbreak risk and the effectiveness of antigen testing.

Our results highlight that the impact of regular antigen testing on the SARS-CoV-2 local outbreak risk is dependent on the regularity of testing, as well as the exact population under consideration (including the level of vaccine- or infection-acquired immunity) and the characteristics of the viral variant responsible for infections. Based on our analyses, we expect antigen testing to reduce the outbreak risk due to the omicron variant but not eliminate it completely. While SARS-CoV-2 is our focus here, our general approach can be applied to other viruses in preparedness for future outbreaks, epidemics, and pandemics beyond COVID-19.

## Results

**Fig. 1.**
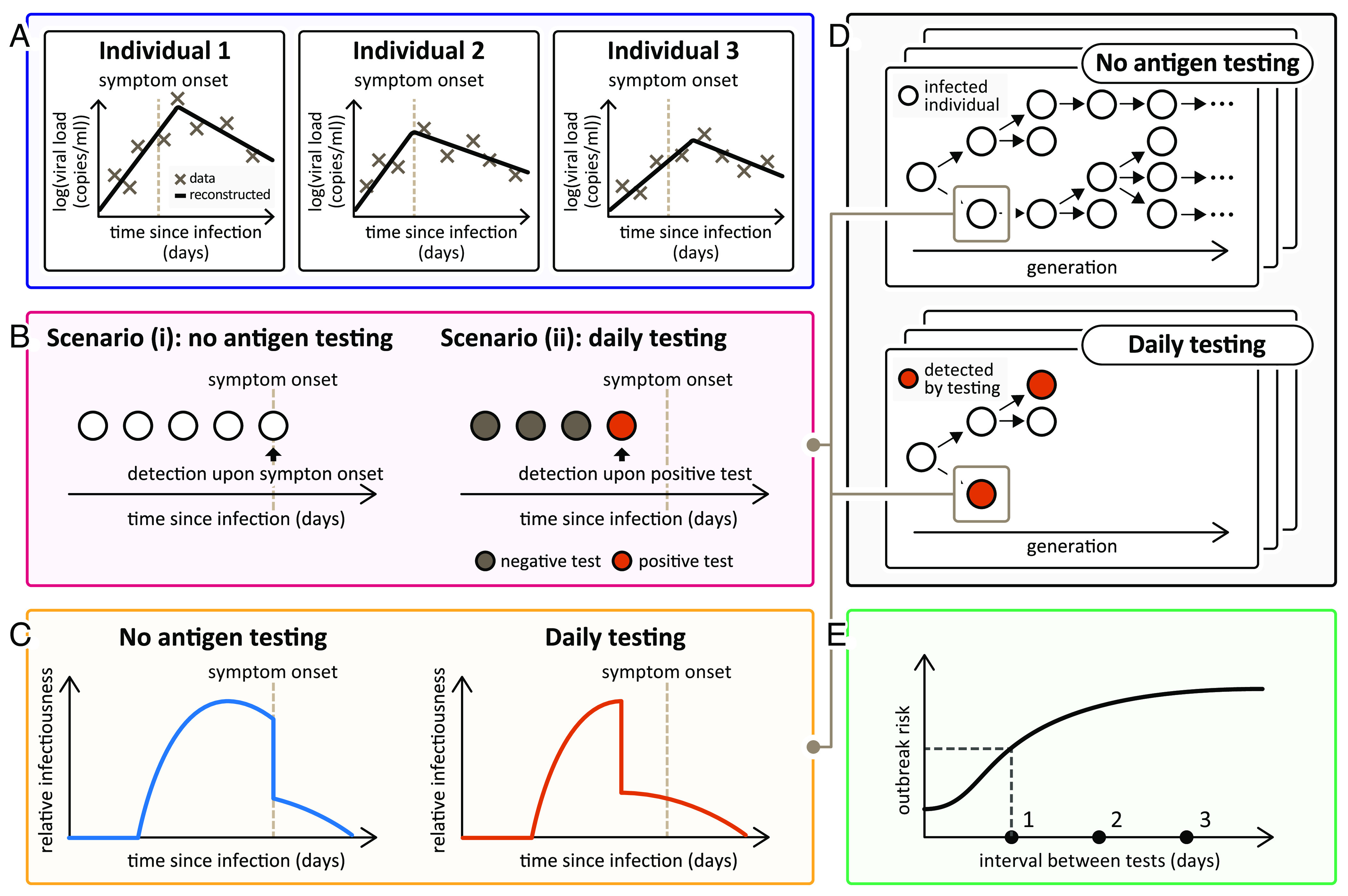
Schematic illustrating our multiscale modelling approach for calculating the SARS-CoV-2 outbreak risk, accounting for regular antigen testing. (*A*) A within-host model is fitted to infection data to infer the temporal viral load profile(s) of infected individuals, potentially accounting for heterogeneity in within-host dynamics between individuals. (*B*) Without antigen testing, infected individuals are detected upon symptom onset [scenario (i)]. Antigen testing facilitates detection before symptoms [scenario (ii)], where the viral load profile(s) inform the probability of a positive test result. (*C*) The viral load profile(s) are used to estimate the infectiousness profile(s) of infected individuals. accounting for a lower transmission risk following detection. Earlier detection under antigen testing leads to a suppressed infectiousness profile. (*D*) Repeated simulation of a stochastic epidemic model incorporating the estimated infectiousness profile(s) can be used to estimate the outbreak risk (the proportion of simulations in which a large outbreak occurs—see *SI Appendix*, Fig. S2). Regular antigen testing breaks chains of transmission, reducing the outbreak risk. (*E*) The outbreak risk can also be derived analytically, allowing the impact of antigen testing to be assessed without large numbers of model simulations.

Our multiscale modelling framework for estimating outbreak risks and analysing the impact of pre-emptive control is outlined in the context of SARS-CoV-2 and regular antigen testing in Fig. 1. In our approach, a within-host model is first fitted to individual infection data to estimate the viral load of infected hosts at each time since infection, potentially considering heterogeneity in within-host dynamics between different individuals (Fig. 1*A*). Accounting for a reduced transmission risk following detection, which may occur prior to symptom onset if regular antigen testing is carried out (Fig. 1*B*), the viral load profile(s) can be used to estimate the infectiousness profile(s) (Fig. 1*C*). The outbreak risk, following a single newly infected individual arriving in an otherwise uninfected population, can then be estimated through repeated simulation of a stochastic epidemic model incorporating the infectiousness profile (Fig. 1*D*). Alternatively, the outbreak risk can be derived analytically—we have derived equations satisfied by the outbreak risk under a branching process transmission model assuming either homogeneous (Eq. **3**) or heterogeneous (*SI Appendix*, Eq. **S5.11**) within-host dynamics between different infected individuals (derivations are given in *SI Appendix*, *Text S5*). The effect on the outbreak risk of factors such as the frequency of antigen testing can then be analysed (Fig. 1*E*).

### The SARS-CoV-2 Local Outbreak Risk and the Impact of Regular Antigen Testing.

**Fig. 2.**
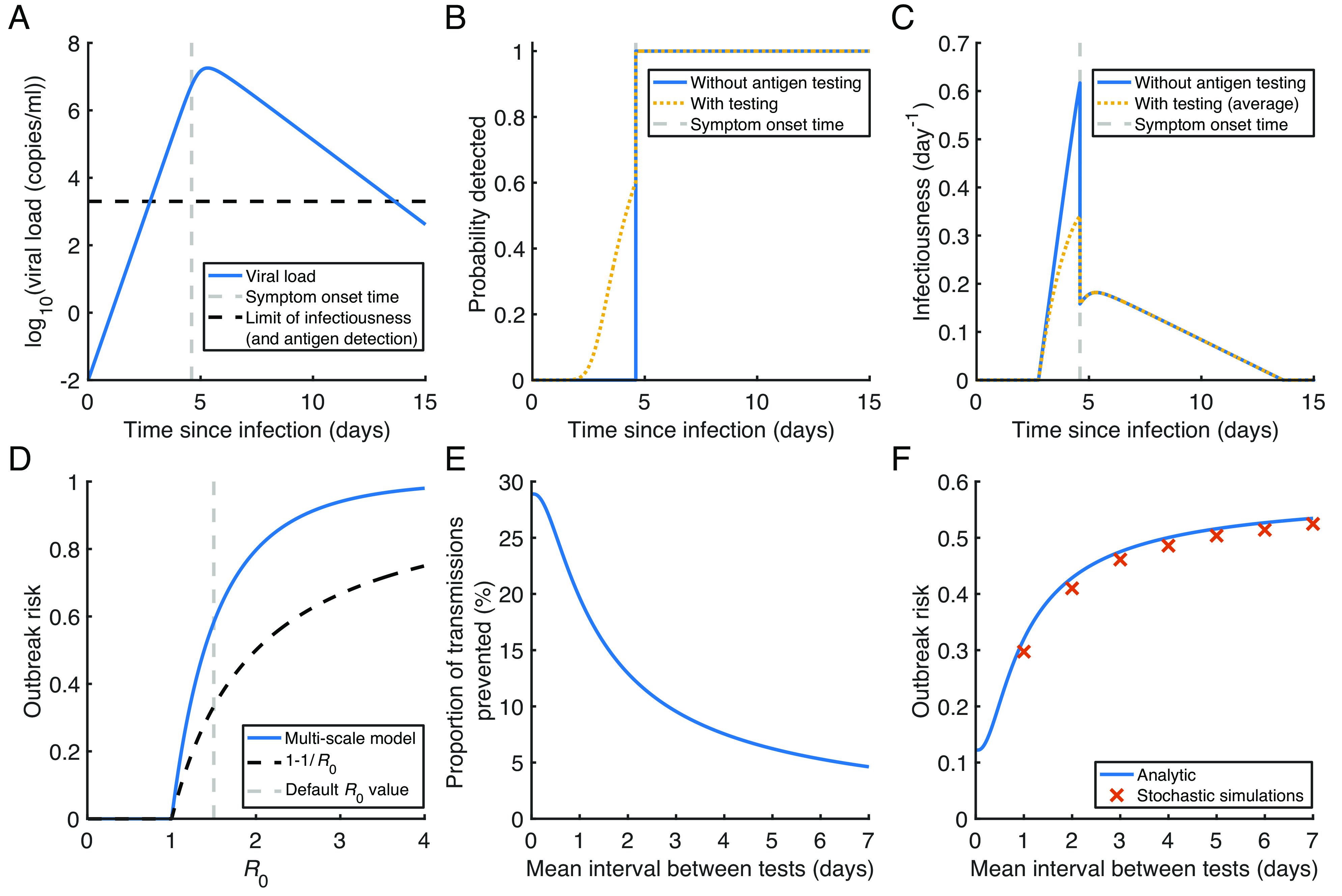
Estimation of the SARS-CoV-2 outbreak risk and analysis of the impact of regular antigen testing. (*A*) Viral load profile using population (median) estimates of within-host model parameters (*SI Appendix*, Table S1). The population incubation period estimate is shown as a vertical grey dashed line, and the assumed viral load threshold for infectiousness and antigen detection is shown as a horizontal black dashed line (we assumed a measurement error affecting antigen test outcomes, leading to the possibility of a positive antigen test with true viral load below this threshold, and vice versa). (*B*) Probability of detection by each time since infection, both without regular antigen testing (blue) and with testing every 2 d (orange dotted), assuming an identical viral load profile and incubation period (as shown in panel *A*) for all infected individuals. (*C*) Infectiousness profiles in the two scenarios, averaging over exact detection times of different individuals in the scenario with antigen testing. (*D*) The probability of a major outbreak without antigen testing for different values of the basic reproduction number for local transmissions, $R_{0}$ , comparing our multiscale approach (blue) with the commonly used formula, $1-1/R_{0}$ (whenever $R_{0}>1$ ; black dashed). (*E*) The proportion of transmissions prevented by regular antigen testing (compared to a scenario where infected individuals are only detected upon symptom onset), $1-R_{0,eff}/R_{0}$ (where $R_{0,eff}$ is the reproduction number accounting for testing), for different values of the mean interval between tests. (*F*) The outbreak risk for different values of the mean interval between tests when $R_{0}=1.5$ in the absence of testing (results for other values of $R_{0}$ are shown in Fig. 3*C*), comparing our analytic multiscale modelling approach (blue) with estimates obtained using a discrete-time stochastic outbreak simulation model (red crosses).

Using nonlinear mixed effects modelling, we fitted a within-host viral dynamics model (41–48) to data from 521 individuals with SARS-CoV-2 omicron variant infections (49). The temporal viral load profile of infected individuals, using population estimates of within-host model parameters (*SI Appendix*, Table S1), is shown in Fig. 2*A*. Model fits to data from individual hosts are shown in *SI Appendix*, Fig. S1.

For simplicity, we initially demonstrated our multiscale modelling approach for estimating the outbreak risk under the assumption of homogeneous within-host dynamics (i.e., we assumed all within-host model parameters, including the incubation period, to be identical for all infected individuals; heterogeneous within-host dynamics are considered later). We used the viral load profile and incubation period in Fig. 2*A* to estimate the probability of detection by each time since infection (Fig. 2*B*), in scenarios without (blue curve) and with (orange dotted curve) regular antigen testing. Without regular antigen testing, we assumed that all infected individuals are detected immediately upon symptom onset. When calculating the detection probability under regular antigen testing, we assumed that the probability of a positive test result is viral load-dependent and that tests are administered at a constant rate (with an exponentially distributed interval between successive tests taken by each individual).

We then estimated the infectiousness profile in each scenario, assuming that infectiousness scales with the logarithm of the viral load (32, 36) above a minimum threshold value and accounting for a reduction in the transmission risk following detection (Fig. 2*C*). When regular antigen testing is carried out, variations in detection times between infected individuals (due to randomness in antigen test timing and outcome) may lead to different individual infectiousness profiles. However, we initially assumed for simplicity that all infected individuals follow the same infectiousness profile, averaged over different possible detection times (using the detection probabilities in Fig. 2*B*).

Local outbreak risk estimates in the absence of regular antigen testing, obtained using either our multiscale modelling approach (using Eq. **3**) or the commonly used population-level estimate, $1-1/R_{0}$ , are shown for a range of values of $R_{0}$ in Fig. 2*D*. Here, and throughout this article, $R_{0}$ denotes the reproduction number for local transmissions at the start of the outbreak without regular antigen testing, accounting for immunity levels within the population and interventions (other than antigen testing) such as social distancing; we denote the reproduction number at the start of the outbreak but accounting for testing (if carried out) by $R_{0,eff}$ . As would be expected, the outbreak risk increases with $R_{0}$ , while our multiscale method generally gives an outbreak risk higher than the standard population-level estimate (when $R_{0}\geq 1$).

**Fig. 3.**
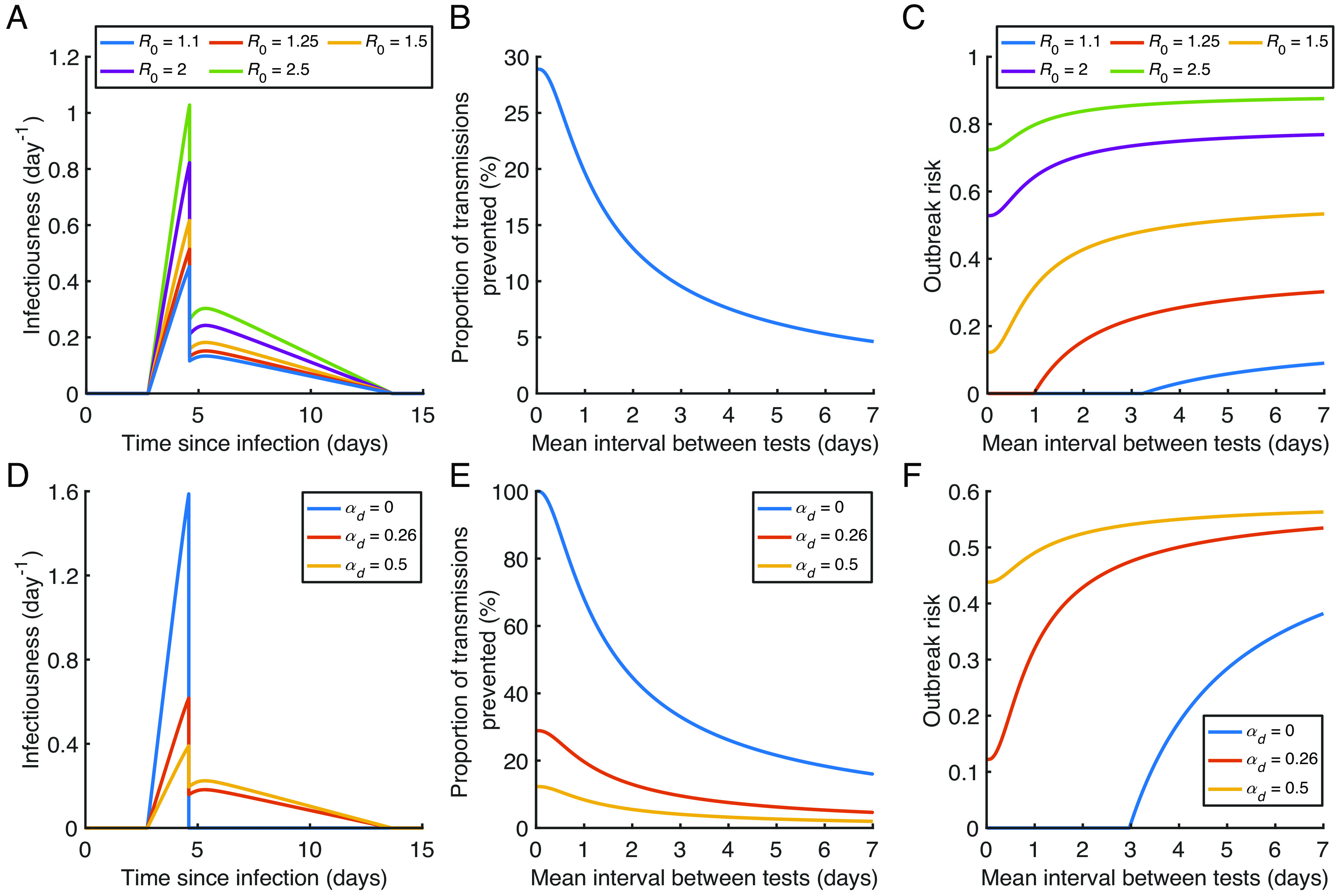
Effect of the local reproduction number and the extent of transmission following detection on the outbreak risk under regular antigen testing. (*A*) Infectiousness profiles without regular antigen testing, for $R_{0}=1.1$ (blue), 1.25 (red), 1.5 (orange), 2 (purple), and 2.5 (green). (*B*) The proportion of transmissions prevented by regular antigen testing for different values of the mean interval between antigen tests (since this quantity is independent of $R_{0}$ , this panel is identical to Fig. 2*E*). (*C*) The outbreak risk for different values of the mean interval between antigen tests, plotted for each $R_{0}$ value. (*D*) Infectiousness profiles without regular antigen testing, assuming the relative infectiousness of a detected host (compared to an undetected individual with the same viral load) is $\alpha_{d}=0$ (blue), 0.26 [red; the value used elsewhere in our analyses (50)], or 0.5 (orange), with $R_{0}=1.5$ in all cases. Under these $\alpha_{d}$ values, the proportions of transmissions that occur prior to symptom onset (without regular antigen testing) are 100%, 39% and 25%, respectively. (*E*) The proportion of transmissions prevented from each infected individual by regular antigen testing, for different values of the mean interval between tests, plotted for each $\alpha_{d}$ value. (*F*) The outbreak risk for different values of the mean interval between tests, plotted for each $\alpha_{d}$ value.

We then used our multiscale model to explore the impact of regular antigen testing on the outbreak risk. First, we estimated the proportion of transmissions prevented from each infected individual, compared to a scenario in which infected individuals are only detected upon symptom onset, under different frequencies of testing (Fig. 2*E*). We then calculated the outbreak risk in each case (Fig. 2*F*), assuming $R_{0}=1.5$ in the absence of testing (different $R_{0}$ values are considered in Fig. 3). Under our baseline model assumptions, daily testing prevents 20% of transmissions (Fig. 2*E*), leading to an outbreak risk of 0.32 (Fig. 2*F*), which is 45% lower than the corresponding outbreak risk without testing (0.58). In comparison, testing every 2 d prevents only 13% of transmissions, giving an outbreak risk of 0.43.

To verify our results, we used a discrete-time, individual-based, stochastic simulation model to estimate the outbreak risk (*SI Appendix*, Fig. S2). We found relatively close agreement between estimates of the outbreak risk under our analytic approach (blue line in Fig. 2*F*) and the stochastic simulations (red crosses).

We investigated the sensitivity of our results to modelling assumptions about detection and infectiousness (*SI Appendix*, Figs. S3 and S4). Compared to our baseline assumption of a log-linear relationship between viral load and infectiousness, an alternative relationship in which infectiousness saturates at high viral loads (37, 51, 52) gives similar outbreak risk estimates under regular antigen testing (*SI Appendix*, Fig. S3*C*). Similarly, explicitly accounting for variations in the detection times of different infected individuals under regular antigen testing (rather than averaging the infectiousness profile over different possible detection times, as in most of our analyses; *SI Appendix*, Fig. S4*A*) has very little effect on the results in Fig. 2*F*. On the other hand, we estimated a lower outbreak risk under daily antigen testing (i.e., antigen testing had a greater impact on the outbreak risk) under the assumption of a constant (fixed) interval between antigen tests, compared to our baseline assumption of a constant (exponential) rate of testing (which was more straightforward to implement in our analytic approach; *SI Appendix*, Fig. S4*B*).

### Effect of the Local Reproduction Number and the Extent of Transmission Following Detection.

In Fig. 2*F*, we considered the outbreak risk under antigen testing for a single value of the basic reproduction number for local transmissions (in the absence of testing), $R_{0}=1.5$ . However, even for SARS-CoV-2, the $R_{0}$ value varies between time periods and local populations because of factors including contact rates, viral evolution, and existing immunity levels. Equivalent results to those in Fig. 2*F* for different $R_{0}$ values are therefore shown in Fig. 3*C*. At $R_{0}$ values of 1.25 or below, daily antigen testing is sufficient to reduce the outbreak risk to zero (by reducing the reproduction number accounting for testing, $R_{0,eff}$ , to below one), whereas the estimated outbreak risk remains high even with very frequent antigen testing for large $R_{0}$ values.

We also explored the effect on our results of the relative transmission risk of a detected host (compared to an undetected host with the same viral load), $\alpha_{d}$ (Fig. 3 *D*–*F*), with a lower $\alpha_{d}$ value corresponding to a higher proportion of presymptomatic transmissions. Whereas in most of our analyses, we assumed a small, but positive, $\alpha_{d}$ value (reflecting that, for example, some household transmission may occur following detection) (50), the blue curves in Fig. 3 *D–F* represent a possibility in which $\alpha_{d}=0$ . This scenario is relevant to populations in which it is possible to completely isolate detected cases from the remainder of the population. In that scenario, very-high-frequency antigen testing can theoretically prevent all transmissions that would otherwise occur (whereas in the remainder of our analyses, only some transmissions can be prevented since transmission can still occur following detection), with testing every 3 d being sufficient to reduce the outbreak risk to zero (when $R_{0}=1.5$).

### Effect of Heterogeneous within-Host Dynamics.

**Fig. 4.**
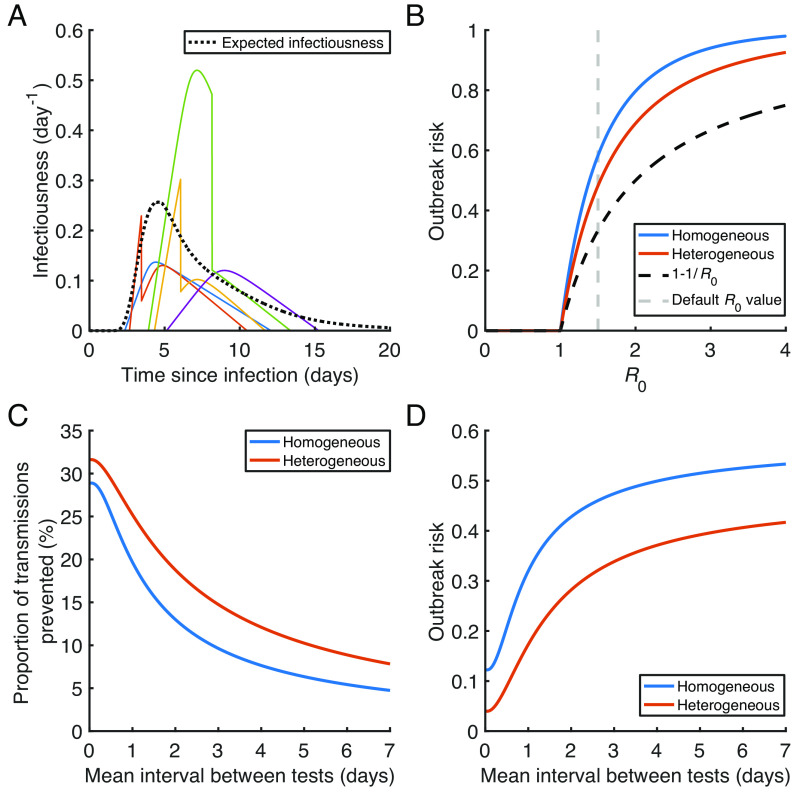
Effect of heterogeneity in within-host dynamics on the outbreak risk under regular antigen testing. (*A*) Example simulated infectiousness profiles for five infected individuals in the absence of regular antigen testing. The profiles were obtained by sampling within-host model parameters using the estimates of fixed and random effects in *SI Appendix*, Tables S1 and S2, respectively. The expected infectiousness profile (obtained by sampling from the random effects 10,000 times, and then taking the average of the resulting individual infectiousness profiles) is shown as a black dotted curve. (*B*) The probability of a major outbreak without antigen testing for different values of $R_{0}$ , comparing our multiscale modelling approach, either assuming homogeneous within-host dynamics (blue) or accounting for heterogeneity between hosts (red), and the commonly used formula, $1-1/R_{0}$ (black dashed). (*C*) The proportion of transmissions prevented by regular antigen testing, for different values of the mean interval between tests, plotted for the models with homogeneous (blue) and heterogeneous (red) within-host dynamics. (*D*) The outbreak risk for different values of the mean interval between tests, for the same scenarios as in panel (*C*).

In order to present our multiscale modelling approach for calculating the outbreak risk in a straightforward setting, up to this point, we considered a scenario of identical within-host viral dynamics for all infected individuals. However, in reality, within-host dynamics differ between individuals. Since we used nonlinear mixed effects modelling to fit our within-host model to viral load data, we were able to estimate the extent of heterogeneity in within-host model parameters between infected individuals (*SI Appendix*, Table S2). We therefore conducted an analysis in which we accounted for such heterogeneity when calculating the localised SARS-CoV-2 outbreak risk (Fig. 4), using the generalised outbreak risk formulation in *SI Appendix*, Eq. **S5.11**.

Without regular antigen testing, we obtained slightly smaller outbreak risk estimates accounting for heterogeneous within-host dynamics, compared to the model with identical within-host dynamics for all infected individuals (Fig. 4*B*). The model with heterogeneous within-host dynamics also gives a higher proportion of transmissions prevented by regular antigen testing (for each testing frequency considered; Fig. 4*C*), contributing to a greater difference in outbreak risk between the two models when regular antigen testing is performed (Fig. 4*D*). For example, the outbreak risk when $R_{0}=1.5$ is 0.48 for the heterogeneous model without testing (0.58 for the homogeneous model), and 0.17 with daily testing (0.32).

### Effect of Asymptomatic Infections.

**Fig. 5.**
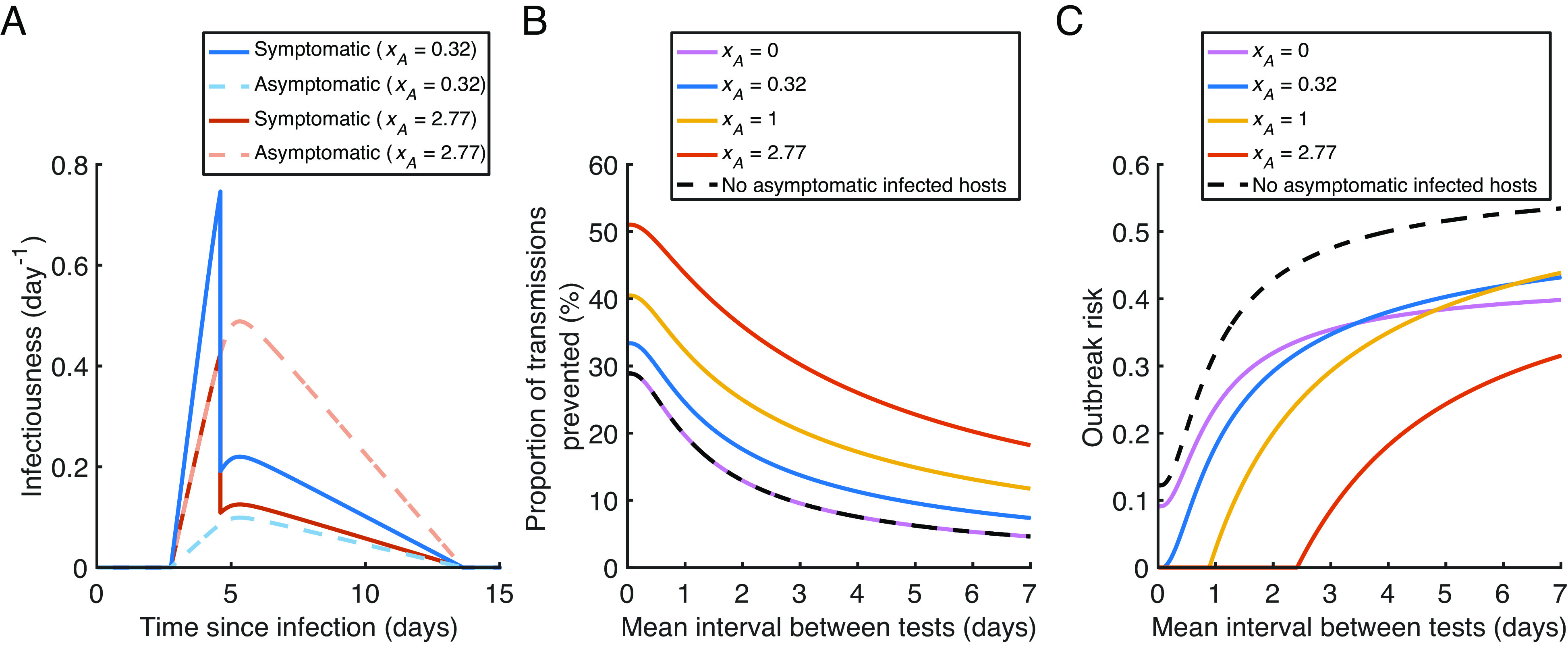
Effect of asymptomatic infections on the outbreak risk under regular antigen testing. (*A*) Infectiousness profiles for infected individuals who develop symptoms (solid lines) and those who remain asymptomatic throughout infection (dashed lines) in the absence of regular antigen testing. We show a scenario in which the average total number of transmissions generated by an entirely asymptomatic infected individual is a factor, $x_{A}=0.32$ , times the corresponding number for a host who develops symptoms (54) (blue), and an alternative possibility where $x_{A}=2.77$ (in this case, presymptomatic and asymptomatic hosts are equally infectious at a given time since infection; red), assuming $R_{0}=1.5$ in both scenarios. (*B*) The proportion of transmissions prevented by regular antigen testing, for different values of the mean interval between tests, accounting for asymptomatic infected hosts with $x_{A}$ values of 0 (pink), 0.32 (blue), 1 (orange) and 2.77 (red), and assuming no asymptomatic infected hosts (black dashed). (*C*) The outbreak risk for different values of the mean interval between tests, for the same scenarios as in panel (*B*).

Using the generalised outbreak risk formulation in *SI Appendix*, Eq. **S5.11**, we also extended our approach to account for entirely asymptomatic infections (Fig. 5), supposing that 25.5% of infected individuals remain without symptoms throughout infection (53) (different proportions of asymptomatic infected hosts are considered in *SI Appendix*, Fig. S5). We assumed that entirely asymptomatic infected individuals remain undetected throughout infection if regular antigen testing is not carried out, and considered a range assumptions regarding the relative infectiousness of entirely asymptomatic infected individuals compared to those who develop symptoms (two possibilities are illustrated in Fig. 5*A*).

In scenarios in which either the relative infectiousness of asymptomatic infected individuals, or the proportion of infected individuals who remain asymptomatic, is higher (so that in both cases, a higher proportion of total transmissions are generated by asymptomatic infected hosts), the outbreak risk with daily antigen testing is lower (Fig. 5*C* and *SI Appendix*, Fig. S5). This is likely because regular antigen testing has the potential to prevent a higher proportion of transmissions from entirely asymptomatic infected hosts (who remain undetected if testing is not carried out) than from those who develop symptoms (Fig. 5*B*).

We note that the assumption that 0% of transmissions are generated by the asymptomatic infected individuals in the population (pink curves in Fig. 5 *B* and *C*) is different from assuming that there are no asymptomatic infected individuals at all (black dashed curves). For example, in the former case, the outbreak risk will be zero whenever the primary infected individual is asymptomatic, whereas in the latter case, the primary infected individual will not remain asymptomatic throughout infection.

### Delayed and/or Time-Limited Antigen Testing.

In most of our analyses of the effect of regular antigen testing on the SARS-CoV-2 local outbreak risk, we focused on a scenario in which testing is in place at the time of virus introduction and continues indefinitely. However, we also generalised our analytic outbreak risk derivation to scenarios where the infectiousness profile is calendar time-dependent (*SI Appendix*, *Text S9*). This enabled us to explore how the effectiveness of antigen testing is reduced if testing is introduced reactively following the first infection occurring (*SI Appendix*, Fig. S6*A*) and/or continues for only a limited time (*SI Appendix*, Fig. S6*B*). We also conducted an analysis in which we assumed a limited number of tests are available to each individual and explored the optimal spacing of these tests to minimise the outbreak risk (for example, 10 tests could be taken daily over 10 d following the first infected individual developing symptoms, or once every 2 d over 20 d; *SI Appendix*, Fig. S6*C*). These analyses therefore demonstrate how antigen-testing strategies can be designed in settings with limited testing resources.

## Discussion

A key challenge for public health policy advisors is estimating the risk that infectious disease cases introduced into a population will lead to a major local outbreak. If the local outbreak risk can be calculated in populations with different characteristics, this enables limited surveillance and control resources to be targeted effectively. In this article, we have presented a modelling framework for estimating the local outbreak risk accounting for within-host viral dynamics.

To demonstrate our multiscale modelling approach in a concrete setting, we focused on the risk of local SARS-CoV-2 outbreaks. We used nonlinear mixed effects modelling to fit a within-host model that has been used extensively to model SARS-CoV-2 viral dynamics (41–48) to data from 521 individuals with omicron variant infections (49). The nonlinear mixed effects approach enabled us to quantify the variability in within-host dynamics between individuals which, in turn, could be used to characterise heterogeneity in individual infectiousness profiles (describing how the transmission risk varies during each infection). We then calculated the local outbreak risk based on these data, assuming either homogeneous or heterogeneous within-host dynamics, and explored the effect of regular antigen testing. We found that regular antigen testing can reduce, but not necessarily eliminate, the outbreak risk, depending on the frequency of testing (for example, in Fig. 2, we estimated an outbreak risk of 0.58 without testing, 0.43 with testing every 2 d, and 0.32 with daily testing) and local transmission characteristics.

Regular antigen testing is an example of an intervention that can be modelled in greater detail using a multiscale modelling approach than is possible using a simpler population-level model. This is because both the probability of a positive test result and the effect of detection on transmission depend on the viral load profile. The timing of testing relative to an individual’s course of infection is therefore crucial for determining the impact of an antigen test. Previous studies have used multiscale models to analyse the effectiveness of antigen testing for controlling an ongoing outbreak (32–34), but none of those studies considered the impact of antigen testing on the outbreak risk.

Antigen testing was carried out at large scale in countries including the United Kingdom (55) earlier in the COVID-19 pandemic, but (similarly to other non-pharmaceutical interventions) has become less commonplace following the rollout of vaccinations. However, local outbreak prevention remains important in some specific populations in the era of living with COVID-19, for example, in care homes due to a high proportion of vulnerable individuals. Our analyses of antigen testing have ongoing relevance to such populations, and the UK government continues to provide free tests to care homes (56).

We expect our qualitative findings regarding important determinants of the effectiveness of regular antigen testing as a pre-emptive intervention to apply widely for SARS-CoV-2 and other viruses. For example, the reproduction number for local SARS-CoV-2 transmissions (accounting for population immunity levels) may be high in specific high-contact settings, or following the emergence of a novel variant associated with increased transmissibility or immune evasion. In such a scenario, the outbreak risk is likely to remain high even under daily testing (Fig. 3*C*). Further mitigations in addition to antigen testing would then be required to substantially reduce the outbreak risk. A similar conclusion likely applies to novel viruses (other than SARS-CoV-2) associated with high transmissibility and against which the population has limited or no immunity. On the other hand, a reduction in the SARS-CoV-2 local reproduction number following a booster vaccination campaign would both reduce the outbreak risk and improve the efficacy of regular antigen testing for reducing any remaining risk (although waning immunity would diminish this effect over time).

For a fixed value of the local reproduction number, we found antigen testing to be more effective when a high proportion of transmissions are presymptomatic (Fig. 3*F*), such as in schools and workplaces (provided symptomatic individuals are instructed to stay at home). This is because population-wide testing enables infected individuals to be detected before symptoms, preventing presymptomatic transmissions that would otherwise have occurred. Similarly, when we accounted for entirely asymptomatic infections, we found a lower outbreak risk under daily testing when a higher proportion of transmissions are generated by asymptomatic infected individuals (Fig. 5*C* and *SI Appendix*, Fig. S5). If the probability of remaining asymptomatic throughout infection is higher for vaccinated infected individuals (53), this may enhance the efficacy of antigen testing for mitigating any residual outbreak risk following a booster vaccination campaign. More generally, because interventions targeting symptomatic individuals and their contacts are likely to be less effective when there is a high proportion of asymptomatic and/or presymptomatic transmissions (57, 58), regular antigen testing may be particularly useful in such scenarios. However, it should be noted that if almost all infections are asymptomatic or mild, it may not be cost-effective for policy makers to implement measures such as antigen testing to reduce the outbreak risk.

In the absence of antigen testing, accounting for heterogeneity in within-host dynamics between different individuals generally gave rise to a lower outbreak risk estimate compared to that obtained under the assumption of homogeneous within-host dynamics. The outbreak risk estimates using both versions of our multiscale modelling approach were higher than a commonly used estimate that does not account for within-host dynamics (19–25) (Fig. 4*B*). These results are consistent with previous comparisons of the outbreak risk between models with different infectious period distributions (26) or offspring distributions (59), although those studies did not consider variations in infectiousness during infection. More variability in the total number of transmissions generated by different individuals typically leads to a lower outbreak risk since, for example, the probability of the primary infected individual generating no transmissions will then be higher. We also found a greater impact of antigen testing on transmission with heterogeneous (as opposed to homogeneous) within-host dynamics (Fig. 4*C*), contributing to a bigger difference in outbreak risk estimates between the heterogeneous and homogeneous models when antigen testing is carried out (Fig. 4*D*) than without testing.

Like any modelling study, our analyses involved assumptions and simplifications. We assumed that infectiousness scales with the logarithm of the viral load (32, 36), with a reduction in the transmission risk upon detection (due to detected individuals staying at home and/or isolating) (50, 60, 61). However, other possibilities could also be considered in the general framework we have developed, as we demonstrated by conducting a supplementary analysis in which infectiousness saturates at high viral loads (37, 51, 52) (*SI Appendix*, Fig. S3). More complex within-host models, or details such as a delay between detection and isolation, would also be straightforward to implement in our multiscale modelling approach. We also assumed equal viral load thresholds for infectiousness and for antigen test positivity, but this assumption could be relaxed, and the effect of antigen test sensitivity [which may vary between tests developed by different manufacturers (62)] on the outbreak risk under testing could be explored. While our focus here was rapid antigen testing, future work may compare the effectiveness of antigen and PCR testing for reducing the outbreak risk, particularly considering a trade-off between test sensitivity and time taken to obtain test results that has previously been explored in the context of controlling an ongoing outbreak (34, 36).

In our analyses, we used data from individuals with SARS-CoV-2 omicron variant infections. The dataset included individuals with a range of infection and vaccination histories (*SI Appendix*, Table S3). As a result, some of the heterogeneity in within-host dynamics that we observed may have been due to differences in prior immunity between different infected individuals. However, in the study originally reporting the data (49), apparently random variations in within-host dynamics were found to be greater than the effects of both immunity and variant (data for other variants in addition to the omicron variant were also considered in ref. 49). Despite this, differences in within-host dynamics between infections due to different SARS-CoV-2 variants, as well as between populations with different levels of pre-existing immunity, may affect quantitative outbreak risk estimates.

Our multiscale modelling approach for estimating the outbreak risk, accounting for heterogeneous within-host viral dynamics, could be extended in numerous directions. We considered a scenario involving a single infected individual arriving in a host population early in their course of infection. However, it would be straightforward to consider possibilities such as the primary infected individual entering the population later in infection, and/or multiple imported cases occurring. A future study may also relate heterogeneity in within-host dynamics to specific characteristics such as age or immunity levels, enabling the outbreak risk to be compared between populations with different structures. Other forms of heterogeneity, such as in susceptibility and/or contact rates, could also be considered. Finally, going forward, we plan to use the mathematical results (*SI Appendix*, *Text S9*) underlying our analysis of reactively introduced antigen testing (*SI Appendix*, Fig. S6) to explore temporal changes in the SARS-CoV-2 local outbreak risk, combining our multiscale modelling approach with previous work incorporating time-dependent susceptibility into outbreak risk estimates (17) (for example, a booster vaccination campaign followed by waning immunity could be considered).

In summary, we have developed a multiscale modelling framework in which within-host viral dynamics models can be used to inform estimates of the risk of infectious disease outbreaks and to analyse the impact of pre-emptive control. Applying our approach to estimate the risk of local SARS-CoV-2 omicron variant outbreaks, we found that regular antigen testing of the local population can reduce, but not eliminate, the outbreak risk, depending on the frequency of testing as well as transmission characteristics that vary temporally and between different populations. Additionally, we found that it is important to consider asymptomatic infection and heterogeneity in within-host dynamics to assess the effectiveness of antigen testing accurately. This research provides an adaptable and widely applicable multiscale modelling framework for guiding pre-emptive control interventions targeting a range of viruses going forward.

## Materials and Methods

### Study Data.

We analysed published viral load data from 521 individuals with symptomatic infections due to the SARS-CoV-2 omicron variant (49). For each individual in the dataset, the results and timing (relative to a recorded symptom onset date, including some tests carried out prior to symptom onset) of at least three RT-qPCR tests were available. The median number of tests per individual was 15. Viral load values (converted from Ct values) were recorded for positive tests.

Viral load data from a randomly chosen subset of 100 individuals are presented in *SI Appendix*, Fig. S1 (along with individual model fits, as described below). Details of the vaccination and previous infection histories of the 521 individuals are given in *SI Appendix*, Table S3.

### Within-Host Model and Parameter Estimation.

We used a simple within-host model of SARS-CoV-2 viral dynamics (41–48), given by

[1] $$\frac{df}{d\tau }=-bfV,$$

[2] $$\frac{dV}{d\tau }=\gamma fV-\delta V,$$

where $f\left(\tau \right)$   and V(τ)   denote, respectively, the proportion of uninfected target cells (so that $f\left(0\right)=1$   ) and viral load at time since infection τ   . This model is a simplified form of a mechanistic within-host model with an additional variable representing infected target cells (63), and can be derived under a quasi-steady state assumption (41, 42) (see *SI Appendix*, *Text S1* for details). The parameters b , γ and δ are the rate constant for virus infection of uninfected cells, the maximum rate constant for viral replication, and the death rate of infected cells, respectively.

We estimated the parameters b , γ and δ by fitting the model to the viral load data. Since symptom onset dates were recorded for all individuals in the dataset, we were also able to estimate the incubation period, $\tau_{inc}$ , assuming an initial viral load value of $V\left(0\right)=0.01$  copies/mL (48). These parameters were estimated using a nonlinear mixed effects modelling approach, amounting to a partial pooling of the data from each individual. This approach both provided overall (population median) parameter estimates (fixed effects; *SI Appendix*, Table S1) and enabled us to characterise variability in parameters between individuals (random effects; *SI Appendix*, Table S2). Individual parameter estimates for each host in the study dataset were also calculated (*SI Appendix*, Fig. S1), although these were not used in our analyses. Details of the parameter estimation approach are given in *SI Appendix*, *Text S2*.

In our initial analyses, we assumed homogeneous within-host dynamics, therefore using only population estimates of the within-host model parameters and the incubation period (the corresponding viral load profile, V(τ) , is shown in Fig. 2*A*). However, we used the random effects estimates when we incorporated heterogeneity in within-host dynamics into our outbreak risk estimation framework (Fig. 4).

### Detection Model.

We assumed that infected individuals could be detected in two ways:

1. By developing symptoms (we assumed previously undetected hosts are detected immediately upon symptom onset).
2. By returning a positive antigen test prior to symptom onset (when regular antigen testing occurs).

Similarly to previous work (46, 48), we assumed that the probability of a positive antigen test result depends on the viral load at the time of testing. Specifically, a positive result was assumed to occur whenever a “measured” viral load, distributed around the true viral load but incorporating a measurement error (normally distributed on the log scale), exceeds the antigen test’s detection limit.

In most of our analyses including regular antigen testing, we assumed a constant rate of testing (with an exponentially distributed interval between tests); the alternative scenario of a constant interval between tests is considered in *SI Appendix*, Fig. S4*B*. Under this assumption, we derived analytically an expression for the probability of an infected individual (with a specified viral load profile) being detected by each time since infection.

Detection probabilities corresponding to the viral load profile and incubation period in Fig. 2*A* (i.e., assuming homogeneous within-host dynamics), both without regular antigen testing and with an average interval of 2 d between tests, are shown in Fig. 2*B*. Details of the detection model are given in *SI Appendix*, *Text S3*, and parameter values are given in *SI Appendix*, Table S1.

### Infectiousness Model.

The infectiousness of each infected host was assumed to scale with the logarithm of their viral load above a minimum threshold value (32, 36), where we assumed that the minimum viral load for infectiousness is equal to the detection limit for antigen testing. An alternative relationship in which infectiousness saturates at high viral loads (37, 51, 52) is considered in *SI Appendix*, Fig. S3. We also accounted for a reduction in (effective) infectiousness following detection, assuming that the infectiousness of a detected host is a constant factor, $\alpha_{d}$ , times that of an undetected host with the same viral load (where $\alpha_{d}$ lies between zero and one).

When regular antigen testing takes place, different infected individuals may be detected at different times since infection, even with homogeneous within-host dynamics (due to randomness in antigen test results and variations in exact testing times). We therefore considered an averaged infectiousness profile under testing, calculated using the detection probabilities described above (this averaging assumption is relaxed in *SI Appendix*, Fig. S4*A*).

Infectiousness profiles corresponding to the viral load profile in Fig. 2*A* and detection probabilities in Fig. 2*B*, both without and with regular antigen testing, are shown in Fig. 2*C*. Details of the infectiousness model are given in *SI Appendix*, *Text S4*, and parameter values are given in *SI Appendix*, Table S1.

### Outbreak Risk.

Here, we outline our approach for calculating the (local) outbreak risk (the probability that a major outbreak results from a single newly infected individual being introduced into an otherwise uninfected population) under the detection and infectiousness models described above. We have derived equations satisfied by the outbreak risk analytically under a branching process transmission model, assuming either homogeneous (Eq. **3**) or heterogeneous (*SI Appendix*, Eq. **S5.11**) within-host dynamics between different infected individuals (derivations are given in *SI Appendix*, *Text S5*). These equations can be solved numerically, avoiding the need to run a large number of stochastic model simulations to estimate the local outbreak risk. However, we also verified our analytically derived outbreak risk estimates against simulations of a discrete-time, individual-based, stochastic epidemic model (Fig. 2*F*; see *SI Appendix*, Fig. S2 and *Text S10* for details).

#### Homogeneous within-host dynamics.

In the simplified scenario where each infected individual follows the same infectiousness profile (i.e., assuming homogeneous within-host dynamics, and averaging over different possible detection times if regular antigen testing is carried out), the outbreak risk, $p_{outbreak}$ , satisfies the implicit equation,

[3] $$p_{outbreak}=1-exp\left(-{R_{0,eff}\times p}_{outbreak}\right),$$

where the largest solution between 0 and 1 should be taken (*SI Appendix*, *Text S5*). Here, $R_{0,eff}$ is the reproduction number at the start of the outbreak (given by the integral of the expected infectiousness profile over all times since infection), accounting for regular antigen testing (assumed to be in place at the time of pathogen introduction) if carried out. In the absence of antigen testing, $R_{0,eff}$ is replaced by the basic reproduction number, $R_{0}$ , in Eq. **3**.

We first solved Eq. **3** numerically in the absence of antigen testing for different values of $R_{0,eff}=R_{0}$ (Fig. 2*D*). For a specified $R_{0}$ value (we considered a baseline value of $R_{0}=1.5$ , but different values are considered in Fig. 3 *A*–*C*), we then used our multiscale modelling approach to determine the impact of regular antigen testing on the (averaged) infectiousness profile and therefore on $R_{0,eff}$ . The proportion of transmissions that are prevented by antigen testing, $1-R_{0,eff}/R_{0}$ , is plotted for different frequencies of testing in Fig. 2*E*. The effect of antigen testing on the outbreak risk could then be analysed (Fig. 2*F*).

#### Heterogeneous within-host dynamics.

In Fig. 4, we considered the impact of heterogeneity in within-host dynamics between different infected individuals on our outbreak risk calculations. In this analysis, we used the random effects estimates characterising individual variations in within-host parameters in *SI Appendix*, Table S2, as well as the generalised equation for the outbreak risk derived in *SI Appendix*, *Text S5* (*SI Appendix*, Eq. **S5.11**). Details of this analysis are given in *SI Appendix*, *Text S7*.

Our generalised outbreak risk formulation accounting for heterogeneity includes as special cases most previous outbreak risk estimates based on branching process approximations of compartmental epidemic models (*SI Appendix*, *Text S6*).

#### Asymptomatic infections.

We conducted an analysis in which we accounted for entirely asymptomatic infected individuals (Fig. 5), assuming that 25.5% of infected hosts remain asymptomatic throughout infection [this value was obtained in a meta-analysis specific to the omicron variant (53); different proportions of asymptomatic infected hosts are considered in *SI Appendix*, Fig. S5]. Asymptomatic infected individuals were assumed to remain undetected throughout infection if regular antigen testing is not carried out (whereas we assumed an immediate drop in the infectiousness of symptomatic hosts following detection upon symptom onset).

For simplicity, in this analysis we assumed identical within-host model parameters for all infected individuals (both those who develop symptoms and those who remain asymptomatic). However, we multiplied the infectiousness profiles of symptomatic and asymptomatic hosts by different constant factors, in order to consider specified values of both $R_{0}$ and the relative overall infectiousness of asymptomatic hosts, $x_{A}$ . Specifically, $x_{A}$ represents the average total number of transmissions generated by an entirely asymptomatic infected individual over the course of infection (in the absence of regular antigen testing), relative to the corresponding quantity for a host who develops symptoms. For example, if $x_{A}=2$ , then each asymptomatic infected individual generates twice as many infections (on average) as each infected individual who develops symptoms. We considered a range of possible $x_{A}$ values in Fig. 5. Further details of this analysis are given in *SI Appendix*, *Text S8*.

### Delayed and/or Time-Limited Regular Antigen Testing.

In most of our analyses, we focused on a scenario in which regular antigen testing is already in place at the time of pathogen introduction and continues indefinitely. However, we also considered scenarios in which testing is introduced reactively after an infection occurs within the local population, and/or testing is only carried out for a limited time period (*SI Appendix*, Fig. S6). An equation for the outbreak risk in these scenarios is derived in *SI Appendix*, *Text S9*.

## Supplementary Material

Appendix 01 (PDF)

## Data Availability

Only previously published data (49) were used in this work. MATLAB code to reproduce our results (compatible with version R2021b) is available at https://github.com/will-s-hart/multiscale-outbreak-risk.
