# Supplementary material for: Analysis of the risk and pre-emptive control of viral outbreaks accounting for within-host dynamics: SARS-CoV-2 as a case study
Source: Proc Natl Acad Sci U S A. 2023 Oct 3;120(41):e2305451120. doi: 10.1073/pnas.2305451120 (PMC10576149; doi:10.1073/pnas.2305451120)
Supplement: Supplementary file 1 — Appendix 01 (PDF) [file pnas.2305451120.sapp.pdf]

PNAS

Supporting Information for

Analysis of the risk and pre-emptive control of viral outbreaks accounting for within-host dynamics: SARS-CoV-2 as a case study

William S Hart[a,b,1], Hyeongki Park[b], Yong Dam Jeong[b,c], Kwang Su Kim[b,d], Raiki Yoshimura[b], Robin N Thompson[a,e,f,2], Shingo Iwami[b,g,h,i,j,k,2]

[a]Mathematical Institute, University of Oxford, Oxford OX2 6GG, United Kingdom
[b]interdisciplinary Biology Laboratory (iBLab), Division of Natural Science, Graduate School of Science, Nagoya University, Nagoya 464-8602, Japan
[c]Department of Mathematics, Pusan National University, Busan 46241, South Korea
[d]Department of Scientific Computing, Pukyong National University, Busan 48513, South Korea
[e]Mathematics Institute, University of Warwick, Coventry CV4 7AL, United Kingdom
[f]Zeeman Institute for Systems Biology and Infectious Disease Epidemiology Research (SBIDER), University of Warwick, Coventry CV4 7AL, United Kingdom
[g]Institute of Mathematics for Industry, Kyushu University, Fukuoka 819-0395, Japan
[h]Institute for the Advanced Study of Human Biology (ASHBi), Kyoto University, Kyoto 606-8501, Japan
[i]Interdisciplinary Theoretical and Mathematical Sciences Program (iTHEMS), RIKEN, Saitama 351-0198, Japan
[j]NEXT-Ganken Program, Japanese Foundation for Cancer Research (JFCR), Tokyo 135-8550, Japan
[k]Science Groove Inc., Fukuoka 810-0041, Japan

[1]Correspondence to: william.hart@maths.ox.ac.uk
[2]These authors contributed equally to this work

This PDF file includes:

Text S1: Derivation of within-host model from target cell-limited model 3

Text S2: Within-host model parameter estimation 4

Text S3: Probability of detection under regular antigen testing 5

Text S4: Details of infectiousness model 7

Text S5: Derivation of outbreak risk 9

Text S6: Wider applicability of generalised outbreak risk formulation 13

Text S7: SARS-CoV-2 local outbreak risk with heterogeneous within-host dynamics 14

Text S8: SARS-CoV-2 local outbreak risk with asymptomatic infections 15

Text S9: Outbreak risk under delayed and/or time-limited regular antigen testing 16

Text S10: Details of discrete-time stochastic outbreak simulation algorithm 18

Table S1: Default parameter values used in our analyses. 22

Table S2: Estimated random effects for within-host model parameters 23

Table S3: Vaccination and previous infection histories of individuals in study dataset 24

Fig. S1: Reconstructed viral dynamics for individual hosts 25

Fig. S2: Alternative estimation of the outbreak risk using a discrete-time, individual-based, stochastic outbreak simulation model 26

Fig. S3: Effect of the relationship between viral load and infectiousness on the outbreak risk under regular antigen testing 27

Fig. S4: Effect of details of implementation of antigen testing in our modelling approach on the outbreak risk under regular antigen testing 28

Fig. S5: Effect of the proportion of asymptomatic infected hosts on the outbreak risk under regular antigen testing 29

Fig. S6: Effect of delayed and/or time-limited antigen testing 30

SI References 31

Text S1: Derivation of within-host model from target cell-limited model

The within-host model used in our analyses (Eqs. 1 and 2 in the main text) can be derived (1,2) from the target cell-limited model (3),

$$\frac{\mathrm{d}T}{\mathrm{d}\tau} = -bTV, \qquad (S1.1)$$

$$\frac{\mathrm{d}I}{\mathrm{d}\tau} = bTV - \delta I, \qquad (S1.2)$$

$$\frac{\mathrm{d}V}{\mathrm{d}\tau} = pI - cV, \qquad (S1.3)$$

where $T(\tau)$, $I(\tau)$ and $V(\tau)$ are the number of uninfected target cells, the number of infected target cells, and the amount of free virus at time since infection τ, respectively. The parameters b, δ and p and c are the rate constant for virus infection, the death rate of infected cells, the viral production rate per infected cell, and the viral clearance rate, respectively.

Under a quasi-steady state assumption (which can be shown to be valid provided c is much larger than δ, as is typically the case for a range of viruses (1)), Eq. S1.3 can be replaced by $0 = pI - cV$. This gives $I(\tau) = cV(\tau)/p$, which can be substituted into Eq. S1.2 in order to obtain an equation in terms of the viral load (on which data were available),

$$\frac{\mathrm{d}V}{\mathrm{d}\tau} = \frac{pb}{c}TV - \delta V. \qquad (S1.4)$$

Finally, letting $T(\tau) = T(0)f(\tau)$, where $f(\tau)$ gives the fraction of target cells that remain uninfected at time since infection τ, in Eqs. S1.1 and S1.4, we recover Eqs. 1 and 2 in the main text, where $\gamma = pbT(0)/c$.

Text S2: Within-host model parameter estimation

Here, we detail how we estimated the parameters in the within-host model (Eqs. 1 and 2 in the main text) by using nonlinear mixed effects modelling to fit the model to individual viral load data (4). Estimated parameters include the within-host model parameters b, γ and δ, in addition to the incubation period, τ_{inc} (estimation of the incubation period amounted to estimating the time of infection of individuals in the study data, relative to recorded symptom onset times). We assumed an initial viral load value of 0.01 copies/ml (5).

In the nonlinear mixed effects model, the value of the individual parameter vector, $\theta_j = (b_j, \gamma_j, \delta_j, \tau_{\mathrm{inc},j})$, for a given host, j, is assumed to be of the form $\theta_j = \theta_{pop} \times e^{\xi_j}$ (where the operations are applied element-wise). Here, θ_{pop} is a fixed effect (also referred to as the population value), and ξ_j is a random effect, assumed to be normally distributed with mean zero and covariance matrix Ω. For simplicity, we assumed the random effects for different parameters to be independent, with standard deviations ω_b, ω_γ, ω_δ and $\omega_{\tau_{\mathrm{inc}}}$ (i.e., $\Omega = \mathrm{diag}(\omega_b^2, \omega_\gamma^2, \omega_\delta^2, \omega_{\tau_{\mathrm{inc}}}^2)$). We estimated both the fixed effects (**Table S1** – note that the subscript *pop* is suppressed) and the standard deviations of the random effects (**Table S2**). Additionally, we estimated the standard deviation, σ, of the measurement error in recorded values of the log viral load (which was assumed to be normally distributed with mean zero).

The Stochastic Approximation of the Expectation-Maximization (SAEM) algorithm (6,7) was used to obtain the parameter values that maximise the likelihood of the recorded viral load data. We accounted for left censoring of viral load data (i.e., a positive test result only occurring when the measured viral load exceeds the detection limit of $10^{2.66}$ copies/ml) in the likelihood. Initial values of estimated parameters were changed multiple times to confirm the robustness of parameter estimation and ensure a global maximum of the likelihood was obtained. We also calculated best-fit estimates (Empirical Bayes Estimates (7)) of within-host model parameters for each individual host (**Fig. S1**). Fitting was implemented in MONOLIX version 2019R2 (7).

Text S3: Probability of detection under regular antigen testing

Supposing that an infected individual conducts an antigen test when their instantaneous viral load is V, we assumed (5,8) that a positive test result occurs with probability $p_+(V) = \mathrm{Prob}(\hat{V} \geq V^*)$. Here, $\hat{V}$ represents a measured viral load, assumed to be normally distributed on the log scale such that $\log_{10}(\hat{V}) \sim N(\log_{10}(V), \sigma^2)$, independently of previous viral load measurements; V^* is the detection limit (the choice of V^* is described in **Table S1**); and the measurement error level, σ, was assumed to be equal to the corresponding quantity that we estimated for PCR testing (**Table S1**). In other words, a positive test result was assumed to occur whenever the measured viral load exceeds the detection limit.

In most of our analyses including regular antigen testing, we assumed an exponentially distributed interval between successive tests with mean T (a constant interval between tests is considered in **Fig. S4B**). Below, we derive (under this assumption) an expression for the probability, $p_d(\tau)$, of an infected individual, subject to regular antigen testing, having been detected by time since infection τ.

First, since we assumed that symptomatic hosts are always detected, we have $p_d(\tau) = 1$ for $\tau \geq \tau_{\mathrm{inc}}$, where τ_{inc} is the individual's incubation period (which can be taken to be infinite to represent an entirely asymptomatic infection). Now, for $\tau < \tau_{\mathrm{inc}}$, we consider a short time interval $[\tau, \tau + \mathrm{d}\tau]$. The probability that the individual returns a positive antigen test in this interval is given (up to terms of order $\mathrm{d}\tau^2$) by $(1/T)\mathrm{d}\tau \times p_+(V(\tau))$, where $(1/T)\mathrm{d}\tau$ represents the probability of taking a test, and $p_+(V(\tau))$ the probability that the result of a test is positive (which depends on the instantaneous viral load, $V(\tau)$). Conditioning on whether or not a positive test is returned in the interval $[\tau, \tau + \mathrm{d}\tau]$ then gives

$$p_d(\tau + \mathrm{d}\tau) = 1 \times \frac{p_+\big(V(\tau)\big)\mathrm{d}\tau}{T} + p_d(\tau) \times \left(1 - \frac{p_+\big(V(\tau)\big)\mathrm{d}\tau}{T}\right), \qquad (\mathrm{S3.1})$$

where the probability of detection by time $(\tau + \mathrm{d}\tau)$ conditional on a positive test in $[\tau, \tau + \mathrm{d}\tau]$ is 1, and the probability conditional on no positive test in that interval is $p_d(\tau)$.

Rearranging the above equation and taking the limit $\mathrm{d}\tau \to 0$ gives the differential equation,

$$\frac{\mathrm{d}p_d}{\mathrm{d}\tau} = \frac{p_+\big(V(\tau)\big)}{T}\big(1 - p_d(\tau)\big), \qquad (\mathrm{S3.2})$$

which can be solved alongside the initial condition $p_d(0) = 0$ to obtain

$$p_d(\tau) = 1 - \exp\left(-\frac{1}{T}\int_0^{\tau} p_+\big(V(x)\big)\mathrm{d}x\right), \tag{S3.3}$$

for $\tau < \tau_{\mathrm{inc}}$.

Text S4: Details of infectiousness model

The infectiousness profile of an undetected host, $\beta_u(\tau)$, at each time since infection, τ, was assumed to depend on their viral load, $V(\tau)$, according to a prescribed functional relationship. Specifically, we assumed (9,10) in most of our analyses that

$$\beta_u(\tau) = K \times \max\{\log_{10}(V(\tau)) - \log_{10}(V^*), 0\}, \quad (S4.1)$$

where the infectiousness limit, V^*, was assumed to be equal to the detection limit for antigen testing. The choice of the scaling factor, K, is described below.

We assumed the effective infectiousness (accounting for behavioural factors) of a detected individual at time since infection τ (where τ exceeds the time of detection) to be a factor α_d times $\beta_u(\tau)$ (the choice of α_d, which lies between zero and one, is described in **Table S1**). In the absence of regular antigen testing, the overall individual infectiousness profile is then

$$\beta(\tau) = \begin{cases} \beta_u(\tau), & \tau < \tau_{\text{inc}}; \\ \alpha_d \beta_u(\tau), & \tau \geq \tau_{\text{inc}}. \end{cases} \quad (S4.2)$$

When regular antigen testing takes place, supposing the individual under consideration has been detected by time since infection τ with probability $p_d(\tau)$, then their expected infectiousness at time since infection τ (accounting for different possible detection times) is

$$\beta(\tau) = [\alpha_d p_d(\tau) + (1 - p_d(\tau))] \times \beta_u(\tau). \quad (S4.3)$$

Assuming that all infected individuals follow the same infectiousness profile, $\beta(\tau)$, the reproduction number, $R_{0,\text{eff}}$, at the start of the outbreak but accounting for regular antigen testing if in place, satisfies

$$R_{0,\text{eff}} = \int_0^\infty \beta(\tau) \mathrm{d}\tau\,. \quad (S4.4)$$

This equation was used to determine the scaling factor, K, in the expression for $\beta_u(\tau)$, under a specified value of the basic reproduction number, $R_{0,\text{eff}} = R_0$, in the absence of regular antigen testing (except where otherwise specified, we took the default value $R_0 = 1.5$). A generalised version of Eq. S4.4 accounting for heterogeneity in the infectiousness profile is given in **Text S5** (Eq. S5.2; this equation was used to calculate K whenever we accounted for such heterogeneity).

In **Fig. S3**, we considered an alternative possibility (11–13) in which Eq. S4.1 is replaced by

$$\beta_u(\tau) = \widetilde{K} \times \frac{V(\tau)^h}{V(\tau)^h + K_m^h}, \quad (S4.5)$$

so that infectiousness saturates at high viral loads. In this case, we took $h = 0.51$ and $K_m = 8.9 \times 10^6$ copies/mL as estimated in (11), while the scaling factor, $\widetilde{K}$, was chosen in the same manner as K in our default infectiousness model.

Text S5: Derivation of outbreak risk

Below, we derive an analytical expression for the outbreak risk in a heterogeneous population divided into n subgroups (the special case of a homogeneous population is obtained when $n = 1$), between which the infectiousness profile of infected hosts (as well as other factors such as susceptibility) may vary. Specifically, we consider a branching process model in which susceptible depletion is neglected and infection lineages are assumed to be independent, and derive the outbreak risk following the introduction of a single newly infected host into the population.

Transmission model

We suppose that each infected host in group j transmits the pathogen to individuals in group i at total rate $\beta_{i,j}(\tau)$ at time since infection τ (a specific form of $\beta_{i,j}(\tau)$ is considered later). The expected total number of infections generated in group i by each infected host in group j (over the course of infection) is then

$$R_{i,j} = \int_0^\infty \beta_{i,j}(\tau)\mathrm{d}\tau\,, \qquad (S5.1)$$

where the basic reproduction number (accounting for regular antigen testing, if in place), $R_{0,\mathrm{eff}}$, is the largest eigenvalue of the matrix with entries $R_{i,j}$ (the next-generation matrix) (14).

We further assume that the transmission rates can be parameterised as $\beta_{i,j}(\tau) = \varepsilon_i \eta_i \beta_j(\tau)$, where $\beta_j(\tau)$ is the infectiousness profile of an infected individual in group j, ε_i is the proportion of the population who are in group i, and η_i is the relative susceptibility in group i (the possibility of heterogeneous susceptibility between different population subgroups is included here for generality, but we did not consider heterogeneous susceptibility in our numerical analyses – i.e., we took $\eta_i = 1$ for each i). Additionally, we define $\bar{\eta} = \sum_{i=1}^{n} \varepsilon_i \eta_i$ to be the average population susceptibility, $a_i = \varepsilon_i \eta_i / \bar{\eta}$ to be the proportion of new infections that are in group i (accounting for both the relative size and susceptibility of each group), B_j to be the total integral of $\beta_j(\tau)$ over all times since infection, and $R_j = \bar{\eta} B_j$ to be the expected total number of

transmissions generated by an infected host in group j (accounting for the susceptibility of the population; note that in all of our numerical analyses, we had $\bar{\eta} = 1$, so that $R_j = B_j$).

Under the above parameterisation, the next generation matrix is of "separable" form $R_{i,j} = \varepsilon_i \eta_i B_j = a_i R_j$, and therefore the basic reproduction number is given by $R_{0,\mathrm{eff}} = \sum_{i=1}^{n} a_i R_i$ (14). Equivalently, this can be written as

$$R_{0,\mathrm{eff}} = \sum_{i=1}^{n} a_i \bar{\eta} B_i = \sum_{i=1}^{n} a_i \bar{\eta} \int_0^\infty \beta_i(\tau) \mathrm{d}\tau = \int_0^\infty \bar{\beta}(\tau) \mathrm{d}\tau, \qquad \text{(S5.2)}$$

where $\bar{\beta}(\tau) = \bar{\eta} \sum_{i=1}^{n} a_i \beta_i(\tau)$ is the expected infectiousness profile (accounting for the susceptibility of the population).

Outbreak risk

Now, we suppose that a single infected individual in group j is introduced into the population at time since infection τ, with the remainder of the population assumed to be uninfected at the time of introduction (and assuming no further external pathogen introductions into the population). An expression for the resulting probability of extinction (i.e., the probability that a major outbreak does *not* occur), denoted $q_j(\tau)$, can be derived by conditioning on whether or not the initial infected individual transmits the pathogen (to an individual in any population group) between times since infection τ and $(\tau + \mathrm{d}\tau)$, to obtain (neglecting the possibility that multiple transmissions occur, which has probability of order $\mathrm{d}\tau^2$)

$$q_j(\tau) = \left(\sum_{i=1}^{n} q_j(\tau + \mathrm{d}\tau) q_i(0) \times \beta_{i,j}(\tau) \mathrm{d}\tau \right) + q_j(\tau + \mathrm{d}\tau) \times \left(1 - \sum_{i=1}^{n} \beta_{i,j}(\tau) \mathrm{d}\tau \right). \quad \text{(S5.3)}$$

Here, $\beta_{i,j}(\tau)\mathrm{d}\tau$ gives the probability of a transmission to an individual in group i occurring in this time interval, $q_j(\tau + \mathrm{d}\tau) q_i(0)$ the extinction probability conditional on such a transmission occurring (since infection lineages are assumed to be independent), and $q_j(\tau + \mathrm{d}\tau)$ the extinction probability conditional on no transmissions occurring.

Rearranging the above equation and taking the limit $\mathrm{d}\tau \to 0$ gives the differential equation,

$$\frac{\mathrm{d}q_j}{\mathrm{d}\tau} = q_j(\tau)\sum_{i=1}^{n}\big(1 - q_i(0)\big)\beta_{i,j}(\tau), \tag{S5.4}$$

which can be solved alongside the boundary condition $q_j(\infty) = 1$ to obtain

$$q_j(\tau) = \exp\left(-\sum_{i=1}^{n}\big(1 - q_i(0)\big)\int_{\tau}^{\infty}\beta_{i,j}(x)\mathrm{d}x\right). \tag{S5.5}$$

In particular, we have

$$q_j(0) = \exp\left(-\sum_{i=1}^{n}\big(1 - q_i(0)\big)R_{i,j}\right). \tag{S5.6}$$

We note that this equation has previously been derived using probability generating functions, rather than using a time-since-infection model as here (15).

Now, under the parameterisation $R_{i,j} = a_i R_j$ (as described above), we have

$$q_j(0) = \exp\left(-R_j\sum_{i=1}^{n}\big(1 - q_i(0)\big)a_i\right). \tag{S5.7}$$

In this case, the overall extinction probability, following the introduction of a single newly infected individual (assuming the initial infection occurs in group j with probability a_j), is

$$q(0) = \sum_{j=1}^{n} a_j q_j(0). \tag{S5.8}$$

Eq. S5.7 above can then be written as

$$q_j(0) = \exp\big(-\big(1 - q(0)\big)R_j\big), \tag{S5.9}$$

and substituting Eq. S5.9 into Eq. S5.8 then gives

$$q(0) = \sum_{j=1}^{n} a_j \exp\big(-\big(1 - q(0)\big)R_j\big). \tag{S5.10}$$

Finally, the outbreak risk (following the introduction of a single newly infected individual into an otherwise susceptible population), $p_{\mathrm{outbreak}} = 1 - q(0)$, then satisfies

$$p_{\mathrm{outbreak}} = 1 - \sum_{j=1}^{n} a_j \times \exp\big(-R_j \times p_{\mathrm{outbreak}}\big). \tag{S5.11}$$

While this equation may have multiple solutions (in particular, $p_{\text{outbreak}} = 0$ is always a solution), by standard theory of hitting probabilities on Markov chains (16), the relevant solution is the largest solution between 0 and 1 (since the relevant solution to Eq. S5.10 is the minimal non-negative one). While we focussed on the outbreak risk starting with a single, newly infected, primary case, our approach could be extended to consider an infected individual introduced into the population later in infection, and/or multiple pathogen introductions.

In the special case of a homogeneous population ($n = 1$), we have

$$p_{\text{outbreak}} = 1 - \exp\left(-R_{0,\text{eff}} \times p_{\text{outbreak}}\right), \qquad (\text{S5.12})$$

i.e., we recover Eq. 3 in the main text. This equation is well-known in the simplified scenario of constant infectiousness during an infectious period of fixed duration (17).

Text S6: Wider applicability of generalised outbreak risk formulation

The result in Eq. S5.11, while derived here in the context of a time-since-infection model in a heterogeneous population, is in fact widely applicable to a range of (branching process) models. Specifically, taking the limit of a continuous distribution of population subgroups in Eq. S5.11 gives the equation

$$p_{\text{outbreak}} = 1 - \int_{\Theta} a(\theta) \times \exp(-R(\theta) \times p_{\text{outbreak}}) \mathrm{d}\theta. \qquad (S6.1)$$

Here $\theta \in \Theta$ is a continuous variable (which may be either real-valued or higher-dimensional) indexing population subgroups and/or possible "types" of infection, $a(\theta)$ is the probability density that a new infection is of type θ, and $R(\theta)$ gives the expected total number of transmissions generated by an infected host with infection type θ. We briefly note that the continuous formulation in Eq. S6.1 is applicable to the scenario of heterogeneous within-host dynamics that we considered in **Fig. 4**, but in practice it was easier to calculate the outbreak risk by sampling the within-host dynamics of a large number of hosts as described in **Text S7**.

As an example to demonstrate the applicability of Eq. S6.1, we consider a branching process approximation of the stochastic SIR compartmental epidemic model. In this case, the possible "types" of infection are indexed by the infectious period, $\theta = t_I \in [0, \infty)$, with $a(\theta) = \mu \exp(-\mu t_I)$ (i.e., an exponentially distributed infectious period is assumed) and $R(\theta) = R_0 \mu t_I$ (i.e., the expected number of transmissions by an infected host is proportional to their infectious period). Substituting into Eq. S6.1 then gives

$$p_{\text{outbreak}} = 1 - \int_0^{\infty} \mu \exp(-(1 + R_0 p_{\text{outbreak}}) \mu t_I) \, \mathrm{d}t_I. \qquad (S6.2)$$

Integrating and taking the largest solution between 0 and 1 of the resulting quadratic equation then reproduces the well-known formula,

$$p_{\text{outbreak}} = \max\left\{1 - \frac{1}{R_0}, 0\right\}. \qquad (S6.3)$$

Similarly, the outbreak risk under branching process approximations of a wide range of more complex compartmental models, for example models with non-exponentially distributed infectious periods and/or age structure, could also be represented using Eq. S6.1.

Text S7: SARS-CoV-2 local outbreak risk with heterogeneous within-host dynamics

To account for heterogeneous within-host dynamics, we used the estimated fixed (**Table S1**) and random (**Table S2**) effects to sample within-host model parameters and incubation periods for $n = 10{,}000$ infected individuals. The infectiousness profile, $\beta_j(\tau)$, of each individual, $j = 1, \dots, n$, was obtained, where this profile was averaged over different possible detection times when analysing regular antigen testing (since this assumption was found to have a very small effect on outbreak risk estimates in **Fig. S4A**). These individual infectiousness profiles were assumed to represent every possible infection pathway in the framework in **Text S5**, each equally likely, so that the outbreak risk, p_{outbreak}, satisfies

$$p_{\text{outbreak}} = 1 - \frac{1}{n}\sum_{j=1}^{n} \exp\left(-R_j \times p_{\text{outbreak}}\right), \qquad (S7.1)$$

(i.e., we took $a_j = 1/n$ for each j), where

$$R_j = \int_0^\infty \beta_j(\tau)\mathrm{d}\tau, \qquad (S7.2)$$

is the individual reproduction number of individual j (accounting for regular antigen testing if carried out).

Text S8: SARS-CoV-2 local outbreak risk with asymptomatic infections

We accounted for entirely asymptomatic infections using the framework in **Text S5** with $n = 2$, with the two population subgroups corresponding to infected individuals who develop symptoms (making up a proportion, $a_1 = 0.745$, of all infected individuals (18)) and those who remain asymptomatic throughout infection (with $a_2 = 0.255$; different proportions of asymptomatic infected hosts are considered in **Fig. S5**). Asymptomatic infected individuals were assumed to remain undetected throughout infection if regular antigen testing is not carried out.

We assumed no difference in within-host model parameters between entirely asymptomatic infected hosts and those who develop symptoms. However, we multiplied the infectiousness profiles of symptomatic and asymptomatic hosts by different constant factors, allowing the expected total number of transmissions generated by an infected host who develops symptoms, R_1, and the corresponding quantity for an asymptomatic infected host, R_2, to be varied independently. Specifically, we chose the R_1 and R_2 values corresponding to specified values of both the basic reproduction number, $R_0 = a_1R_1 + a_2R_2$, and the relative overall transmissibility of asymptomatic infected hosts, $x_A = R_2/R_1$, in the absence of regular antigen testing.

We considered x_A values of 0 (so that some infected individuals are entirely asymptomatic, but these individuals generate no transmissions), 0.32 (the central estimate obtained in a meta-analysis (19) conducted using data up to July 2021, prior to the emergence of the omicron variant), 1 and 2.77 (corresponding to a scenario where all undetected individuals are equally infectious at a given time since infection, regardless of whether or not they go on to develop symptoms).

In this framework, the proportion of all transmissions arising from entirely asymptomatic infectors (in the absence of regular antigen testing) is given by

$$r_A = \frac{a_2R_2}{a_1R_1 + a_2R_2} = \frac{a_2x_A}{a_1 + a_2x_A}. \qquad (S8.1)$$

The x_A values of 0, 0.32, 1 and 2.77 (with $a_2 = 0.255$) give values of the percentage of total transmissions that are generated by asymptomatic hosts (without antigen testing) of 0%, 10%, 26% and 49%, respectively.

Text S9: Outbreak risk under delayed and/or time-limited regular antigen testing

Here, we generalise our results to obtain an expression for the outbreak risk in scenarios where regular antigen testing is introduced reactively after an infection occurs and/or is only in place for a limited period of time. For simplicity, we consider homogeneous within-host dynamics (although the derivation presented here readily generalises to a heterogeneous population), supposing that each host infected at calendar time t transmits the pathogen at rate $\beta(\tau, t)$ at time since infection τ (i.e., at calendar time $(t+\tau)$). Below, we first derive the outbreak risk for general $\beta(\tau, t)$, before deriving a specific form of $\beta(\tau, t)$ under delayed and/or time-limited regular antigen testing.

We suppose that an individual, who was infected at calendar time t, is introduced into an otherwise uninfected population at time since infection τ (at calendar time $(t+\tau)$). Then, conditioning on whether or not the initial infected host transmits the pathogen between times since infection τ and $(\tau + \mathrm{d}\tau)$ (and assuming no more external infections), we find that the extinction probability, $q(\tau, t)$, satisfies (up to terms of order $\mathrm{d}\tau^2$)

$$q(\tau, t) = q(\tau + \mathrm{d}\tau, t) q(0, t + \tau + \mathrm{d}\tau) \times \beta(\tau, t)\mathrm{d}\tau + q(\tau + \mathrm{d}\tau, t) \times (1 - \beta(\tau, t)\mathrm{d}\tau). \quad \text{(S9.1)}$$

Rearranging and taking the limit $\mathrm{d}\tau \to 0$ gives the differential equation,

$$\frac{\partial q}{\partial \tau} = q(\tau, t)\big(1 - q(0, t + \tau)\big)\beta(\tau, t), \quad \text{(S9.2)}$$

which can be solved alongside the boundary condition $q(\infty, t) = 1$ to obtain

$$q(\tau, t) = \exp\left(-\int_{\tau}^{\infty} \big(1 - q(0, t + \tilde{\tau})\big)\beta(\tilde{\tau}, t)\mathrm{d}\tilde{\tau}\right). \quad \text{(S9.3)}$$

In particular, we have

$$q(0, t) = \exp\left(-\int_{0}^{\infty} \big(1 - q(0, t + \tau)\big)\beta(\tau, t)\mathrm{d}\tau\right), \quad \text{(S9.4)}$$

(where we have relabelled $\tilde{\tau}$ from the previous equation as τ). The outbreak risk, $p_{\mathrm{outbreak}}(t) = 1 - q(0, t)$, following the introduction of a single newly infected host at time t, is therefore the largest solution between 0 and 1 of the equation

$$p_{\mathrm{outbreak}}(t) = 1 - \exp\left(-\int_{0}^{\infty} p_{\mathrm{outbreak}}(t + \tau)\beta(\tau, t)\mathrm{d}\tau\right). \quad \text{(S9.5)}$$

We now derive the form of $\beta(\tau, t)$ under delayed and/or time-limited regular antigen testing. Specifically, we suppose that testing only takes place between calendar times t_{start} and t_{end}, and that within that time period, the interval between

tests (for a specified individual) is exponentially distributed with mean T. A similar argument to that in **Text S3** can be used to show that the probability, $p_d(\tau, t)$, of an individual infected at calendar time t having been detected by time since infection $\tau < \tau_{\mathrm{inc}}$ (where τ_{inc} is the incubation period, with $p_d(\tau, t) = 1$ for $\tau \geq \tau_{\mathrm{inc}}$), is

$$p_d(\tau, t) = 1 - \exp\left(-\frac{1}{T}\int_{\max\{0,\, \min\{\tau,\, t_{\mathrm{start}} - t\}\}}^{\max\{0,\, \min\{\tau,\, t_{\mathrm{end}} - t\}\}} p_+\big(V(x)\big)\mathrm{d}x\right), \qquad (S9.6)$$

where $p_+(V(x))$ gives the probability that the result of a test taken at time since infection x is positive, and the limits of the integral give the times since infection up to τ over which the regular antigen testing policy is in place. The (calendar time-dependent) expected infectiousness profile, accounting for different possible detection times, is then

$$\beta(\tau, t) = \left[\alpha_d p_d(\tau, t) + \big(1 - p_d(\tau, t)\big)\right] \times \beta_u(\tau), \qquad (S9.7)$$

where $\beta_u(\tau)$ is the infectiousness profile of an undetected individual at time since infection τ, and α_d is the relative infectiousness of a detected host.

Finally, we consider a scenario in which regular antigen testing is introduced after a delay of x_{del} from the time of the first infection (where we may expect x_{del} to be at least the length of the incubation period), and is carried out over a finite duration of time, x_{dur}. This scenario can be represented by taking $t_{\mathrm{start}} = 0$ and $t_{\mathrm{end}} = x_{\mathrm{dur}}$ in the above, and then using Eq. S9.5 to calculate $p_{\mathrm{outbreak}}(-x_{\mathrm{del}})$ numerically. In this scenario, for $t \geq t_{\mathrm{end}}$ (i.e., after antigen testing has ended), $p_{\mathrm{outbreak}}(t)$ is independent of t and can be calculated using Eq. 3 in the main text. Eq. S9.5 can then be solved iteratively on a grid of $t \in [-x_{\mathrm{del}}, t_{\mathrm{end}}]$ by considering successively lower t values and each time discretising the integral in Eq. S9.5 to calculate $p_{\mathrm{outbreak}}(t)$, since Eq. S9.5 allows $p_{\mathrm{outbreak}}(t)$ to be calculated once $p_{\mathrm{outbreak}}(x)$ is known for all $x > t$.

Text S10: Details of discrete-time stochastic outbreak simulation algorithm

We verified our analytically derived estimates of the outbreak risk by comparing these values with corresponding estimates obtained through repeated simulation of a discrete-time, individual-based stochastic epidemic model (**Fig. 2F** and **Fig. S2**). In this section, we describe the simulation model and how it was used to estimate the outbreak risk.

Prior to running each outbreak simulation, we first determined and discretised the within-host dynamics that each individual, i, in the population would follow if ever infected, according to the following steps:

1. Determine the individual's (continuous-time) viral load profile, $V^{(i)}(\tau)$, where τ is the time since infection, and their incubation period, $\tau_{\text{inc}}^{(i)}$ (in **Fig. 2F** and **Fig. S2,** we assumed homogeneous within-host dynamics, but in principle heterogeneity could be included).
2. Calculate the individual's undetected infectiousness profile, $\beta_u^{(i)}(\tau)$, and probability of antigen test positivity, $p_+^{(i)}(\tau)$, as described in the main text (note that $p_+^{(i)}$ is here defined as a function of time since infection, τ, rather than viral load).
3. Sample the (potential) time, $r^{(i)}$, from the start of the day of infection to the exact (potential) infection time, uniformly between zero days and one day.
4. For each day since infection, $\tau_{\text{discr}} \geq 1$ (where τ_{discr} is integer-valued and the day of infection is denoted day 0), calculate the discretised undetected infectiousness, $\beta_{u,\text{discr}}^{(i)}(\tau_{\text{discr}})$, as the average value of $\beta_u^{(i)}(\tau)$ between times since infection $(\tau_{\text{discr}} - r_i)$ and $(\tau_{\text{discr}} - r_i + 1)$. Note that implicit in our simulation algorithm is the assumption that hosts cannot transmit the pathogen on the day of infection (i.e., $\beta_{u,discr}^{(i)}(0) = 0$).
5. Calculate the probability, $p_{+,\text{discr}}^{(i)}(\tau_{\text{discr}}) = p_+^{(i)}(\tau_{\text{discr}} - r_i)$, of a test taken at the start of day of infection $\tau_{\text{discr}} \geq 1$ giving a positive result.
6. Calculate the discretised incubation period, $\tau_{\text{inc,discr}}^{(i)} = \left\lfloor \left(r^{(i)} + \tau_{\text{inc}}^{(i)} \right) \right\rfloor$. Note that we only considered a single continuous incubation period, which exceeded 1, but if a non-trivial distribution is used, then it should be truncated to take values of at least 1 in order to avoid symptom onset occurring on the day of infection.

7. Calculate the relative infectiousness on the day of symptom onset (assuming the host is not detected before developing symptoms), $\alpha_o^{(i)}$ (so that the individual's infectiousness on the day of symptom onset is $\alpha_o^{(i)}\beta_{u,\mathrm{discr}}^{(i)}(\tau_{\mathrm{inc,discr}}^{(i)})$), chosen to ensure that the continuous- and discrete-time infectiousness profiles give the same expected number of transmissions during this day (under the assumption of isolation immediately following the exact symptom onset time).
8. Calculate the total duration of infection (up to loss of infectiousness), $\tau_{\mathrm{rec,discr}}^{(i)}$, as the earliest day of infection for which $\beta_{u,\mathrm{discr}}^{(i)}(\tau_{\mathrm{discr}}) = 0$ for all $\tau_{\mathrm{discr}} \geq \tau_{\mathrm{rec,discr}}^{(i)}$.

An example discretised infectiousness profile (without regular antigen testing) is shown in **Fig. S2A**.

In the simulation algorithm, individuals are classified as being in one of the following states on each day: susceptible (S), infected but undetected (U), infected with symptom onset on the current day (and not detected prior to onset; O), infected and detected (D), or recovered (specifically, no longer infectious following an infection; R). The O stage is included to allow for symptom onset (and therefore detection) occurring at any time of day, whereas for simplicity we assumed that regular antigen testing takes place only at the start of each day. The status of individual i (at a given step in the simulation) is denoted by $Y^{(i)} \in \{S, U, O, D, R\}$. We write, for example, $\mathbf{1}_S(Y^{(i)})$, to denote the indicator function that takes the value one if $Y^{(i)} = S$, and zero otherwise. However, we emphasise that the simulation model is not a compartmental model, since different individuals in the same state are not treated identically.

Now, the simulation algorithm has the following inputs:

- The population size, N.
- The relative infectiousness of detected hosts, α_d.
- The relative susceptibility, $\eta^{(i)}$, of each individual, i (note that we assumed homogeneous susceptibility in our analyses, i.e., $\eta^{(i)} = 1$ for each i).
- The quantities $\beta_{\mathrm{u,discr}}^{(i)}(\tau_{\mathrm{discr}})$, $p_{+,\mathrm{discr}}^{(i)}(\tau_{\mathrm{discr}})$, $\tau_{\mathrm{inc,discr}}^{(i)}$, $\alpha_o^{(i)}$ and $\tau_{\mathrm{rec,discr}}^{(i)}$, which characterise discretised individual within-host dynamics (as described above).
- The number of antigen tests, $Z^{(i)}(t)$, conducted by individual i at the start of day t of the simulation (for each positive integer value of t). We considered two possibilities:

i. In **Fig. 2F**, we sampled $Z^{(i)}(t)$ from a Poisson distribution with mean $1/T$ (independently for each individual and each day, where a range of T values were considered). This is consistent with our analytic outbreak risk derivation (since an exponentially distributed duration between tests, with mean T (measured in days), leads to a Poisson-distributed number of tests being taken each day, with mean $1/T$, although we note that tests may be taken at any time of day in the analytic approach), but leads to the possibility of more than one daily test.

ii. In **Fig. S4B**, we additionally considered a fixed (constant) gap of length T between days on which a test is taken. For each individual, we sampled the first day of the simulation on which a test is conducted uniformly between 1 and T (independently for different individuals).

The outbreak simulation algorithm consists of the following steps:

1. Initialise the time at $t = 0$ days and the status of each host at $Y^{(i)} = S$.
2. Sample a single initial infected host, i, according to the relative susceptibilities, η_j (i.e., host j is selected with probability $\eta_j / \sum_{k=1}^{N} \eta_k$). Set $Y^{(i)} = U$ and the infection time, $t_{\text{inf}}^{(i)} = 0$.
3. While $\sum_{i=1}^{n} \mathbf{1}_{\{U,O,D\}}\left(Y^{(i)}\right) > 0$ (i.e., while the number of active infections is greater than zero), repeat the following steps:
   a. Increase the simulation time, t, by 1 day (i.e., set $t = (t + 1)$).
   b. For each i such that both $Y^{(i)} \in \{U, O, D\}$ and $\left(t_{\text{inf}}^{(i)} + \tau_{\text{rec,discr}}^{(i)}\right) = t$, set $Y^{(i)} = R$ (recovery/loss of infectiousness).
   c. For each i such that $Y^{(i)} = O$, set $Y^{(i)} = D$ (day after symptom onset).
   d. For each i such that $Y^{(i)} = U$, carry out the following steps (testing process):
      i. Generate a random number, r, uniformly distributed between 0 and 1.
      ii. If $r < 1 - \left(1 - p_{+,\text{discr}}^{(i)}\left(t - t_{inf}^{(i)}\right)\right)^{Z^{(i)}(t)}$, set $Y^{(i)} = D$.
   e. For each i such that both $Y^{(i)} = U$ and $\left(t_{\text{inf}}^{(i)} + \tau_{\text{inc,discr}}^{(i)}\right) = t$, set $Y^{(i)} = O$ (symptom onset).
   f. Calculate the total infectious pressure exerted on each susceptible individual over the current simulation day,

$$\lambda(t) = \frac{1}{N}\left(\sum_{j=1}^{N}\left(\mathbf{1}_U\left(Y^{(j)}\right) + \alpha_o^{(j)}\mathbf{1}_O\left(Y^{(j)}\right) + \alpha_d\mathbf{1}_D\left(Y^{(j)}\right)\right)\beta_{\mathrm{u,discr}}^{(j)}\left(t - t_{inf}^{(j)}\right)\right). \quad \text{(S10.1)}$$

g. For each i such that $Y^{(i)} = S$, carry out the following steps (transmission process):

   i. Generate a random number, r, uniformly distributed between 0 and 1.

   ii. If $r < 1 - \exp\left(-\eta^{(i)}\lambda(t)\right)$, set $Y^{(i)} = U$ and $t_{inf}^{(i)} = t$.

For each testing scenario considered, we carried out 100,000 model simulations in a population of $N = 1{,}000$ individuals. The outbreak risk was estimated as the proportion of model simulations in which the total number of individuals ever infected exceeded 10% of the total population (see **Fig. S2**).

Table S1

Parameters	Symbol	Unit	Value	How obtained
Rate constant for virus infection	b	(copies/ml) $^{-1}$ day^{-1}	1.43×10^{-7}	Fitted to viral load data
Maximum rate constant for viral replication	γ	day^{-1}	5.64	Fitted to viral load data
Death rate of infected cells	δ	day^{-1}	1.21	Fitted to viral load data
Incubation period	τ_{inc}	days	4.60	Fitted to viral load data
Initial quantity of free virus	$V(0)$	copies/ml	0.01	Assumed (5)
Standard deviation of error in log viral load measurements	σ	$\log_{10}$ (copies/ml)	0.87	Fitted to viral load data
Viral load limit of infectiousness and antigen test positivity	V^*	copies/ml	2.00×10^3	Minimum viral load for culturable virus for the omicron variant from (20)
Relative infectiousness of detected hosts	α_{d}	---	0.26	Estimated value for the delta variant from (21) (other values considered in **Fig. 3**)
Reproduction number at time of introduction in absence of regular antigen testing	R_0	---	1.5	Assumed (other values considered in **Fig. 3** and elsewhere)
Mean interval between antigen tests when regular testing conducted	T	days	2	Assumed (**Fig. 2BC** only; a range of values considered elsewhere)

Table S1: Default parameter values used in our analyses. The values given here for parameters in our within-host, detection and infectiousness models were used in our analyses except where explicitly stated otherwise. Note that the values of the within-host model parameters b, γ, δ and τ_{inc} here are population median estimates (fixed effects); estimates of random effects are given in **Table S2**.

Table S2

Parameters	Symbol	Value
Random effect for rate constant for virus infection	ω_b	1.33
Random effect for maximum rate constant for viral replication	ω_γ	0.15
Random effect for death rate of infected cells	ω_δ	0.54
Random effect for incubation period	$\omega_{\tau_{\mathrm{inc}}}$	0.29

Table S2: Estimated random effects for within-host model parameters. The estimated quantities correspond to the standard deviation between different infected hosts of individual values of the natural logarithm of the parameters b, γ, δ and τ_{inc} (see the section "Within-host model and parameter estimation" of **Materials and Methods** in the main text for details; population parameter values (fixed effects) and units are given in **Table S1**).

Table S3

Characteristic	Status	Number of individuals
Vaccination information	Unvaccinated	7
	Fully vaccinated (not boosted)	136
	Boosted	378
Previous infection history	No previous infection	443
	Previous infection	78

Table S3: Vaccination and previous infection histories of individuals in study dataset. We used published viral load data from 521 individuals with SARS-CoV-2 omicron variant infections (4) in our analyses. Here, the COVID-19 vaccination and previous SARS-CoV-2 infection (i.e., prior to the infection that was considered in the study dataset) histories of these 521 individuals are summarised. Individuals labelled as fully vaccinated (not boosted) had received (at the time of latest PCR data collection) either two doses of an mRNA vaccine, or one dose of the Janssen (Ad.26.COV2.S) adenovirus vector-based vaccine, while individuals labelled as boosted had received an additional mRNA vaccine dose.

Fig. S1

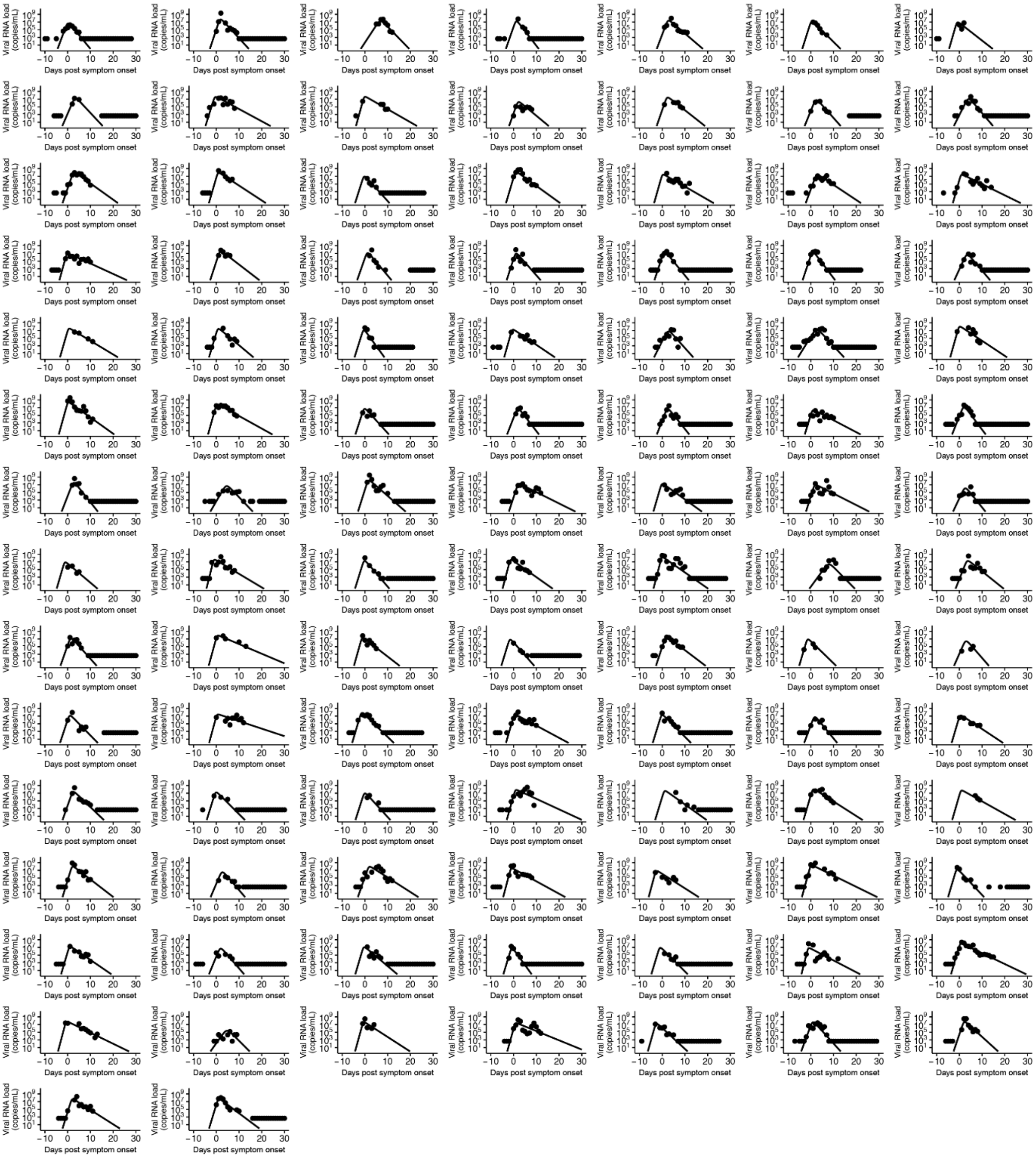

Fig. S1: Reconstructed viral dynamics for individual hosts. Individual-level within-host model fits to longitudinal SARS-CoV-2 viral load data are shown (see the section "Within-host model and parameter estimation" of Materials and Methods in the main text). Overall, we used data from 521 individuals with SARS-CoV-2 omicron variant infections (4); here, individual model fits are shown for 100 randomly chosen individuals. In each panel (corresponding to a single individual), the dots indicate the measured viral load data, and the solid curves give the estimated viral load at different times relative to symptom onset.

Fig. S2

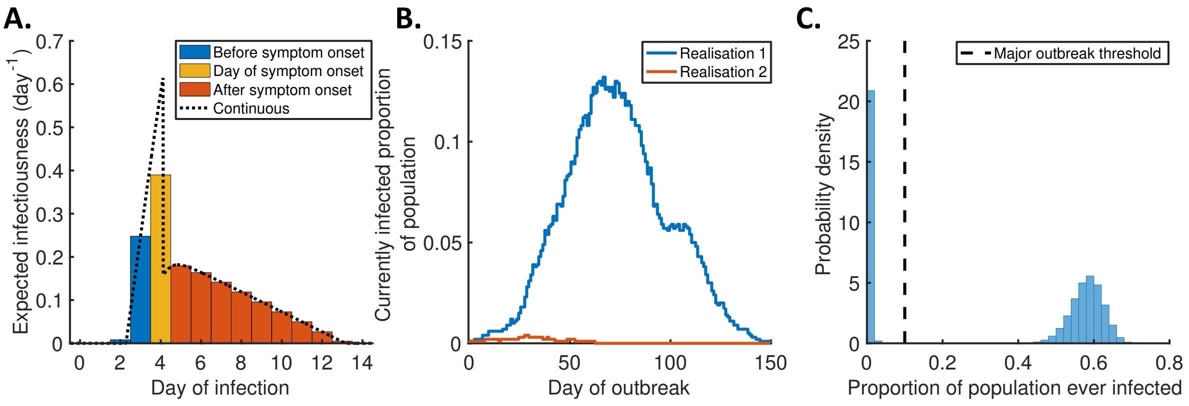

Fig. S2: Alternative estimation of the outbreak risk using a discrete-time, individual-based, stochastic outbreak simulation model. A. Example discretised infectiousness profile of a single infected host when regular antigen testing does not take place. **B**. The output of two realisations of the stochastic outbreak simulation model. **C**. Histogram of total outbreak sizes (i.e., the total proportion of the population ever infected during the outbreak) over 100,000 model simulations. The vertical black dashed line indicates the assumed threshold for a major outbreak of 10% of the population being infected. Here (without regular antigen testing), the estimated outbreak risk (i.e., the proportion of model simulations classified as major outbreaks) is 0.58. Details of the stochastic simulation model are given in **Text S10**.

Fig. S3

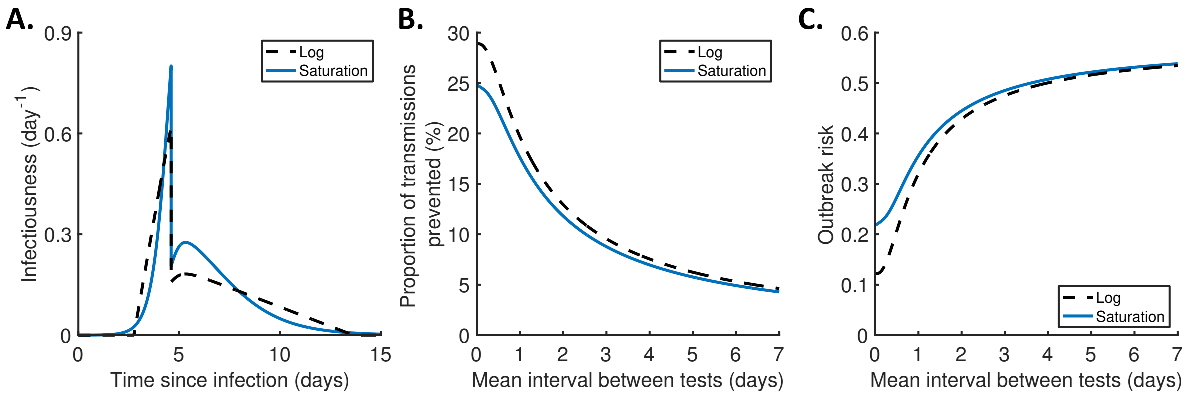

Fig. S3: Effect of the relationship between viral load and infectiousness on the outbreak risk under regular antigen testing. A. Infectiousness profiles without regular antigen testing, assuming infectiousness either scales with the logarithm of the viral load (9,10) (as in our main analyses; black dashed) or saturates at high viral loads (blue), with $R_0 = 1.5$ in both cases. Details of the two infectiousness models are given in **Text S4** (11–13). **B**. The proportion of transmissions prevented by regular antigen testing, for different values of the mean interval between antigen tests, under the two relationships between viral load and infectiousness. **C**. The outbreak risk for different values of the mean interval between antigen tests under the two relationships.

Fig. S4

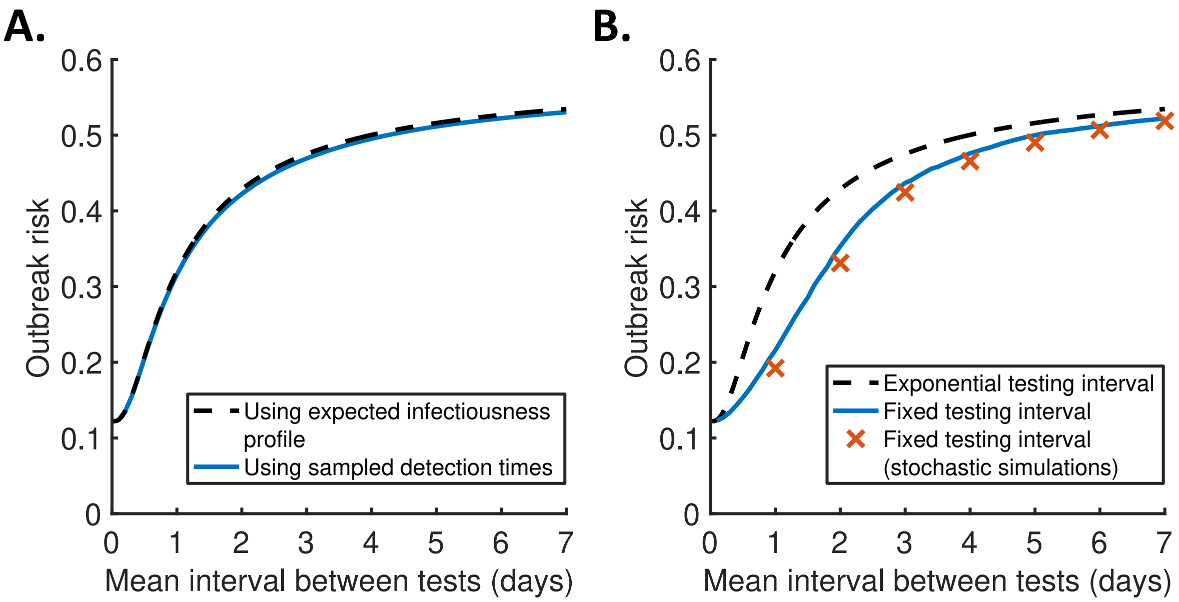

Fig. S4: Effect of details of implementation of antigen testing in our modelling approach on the outbreak risk under regular antigen testing. A. The outbreak risk for different values of the mean interval between antigen tests, comparing our default analytic approach using an expected infectiousness profile that averages over the individual infectiousness profiles of hosts with different detection times (blue), and a more complex approach in which variations in detection times are accounted for directly (red dashed). Variable detection times were accounted for by sampling the detection times of 10,000 infected individuals and using Eq. S5.11 to calculate the outbreak risk (assuming the resulting infectiousness profiles of the 10,000 hosts correspond to equally likely possible infection pathways, similarly to how we accounted for heterogeneous within-host dynamics in **Text S7**). **B.** The outbreak risk for different values of the mean interval between tests, comparing our default analytic approach with an exponentially distributed interval between tests (black dashed), and both the analytic (blue) and simulation-based approaches (red crosses) under the alternative assumption of a fixed (constant) interval between tests. Note that in the analytic approach with a fixed interval, we used sampled detection times since the expected infectiousness profile was not readily available in this case.

Fig. S5

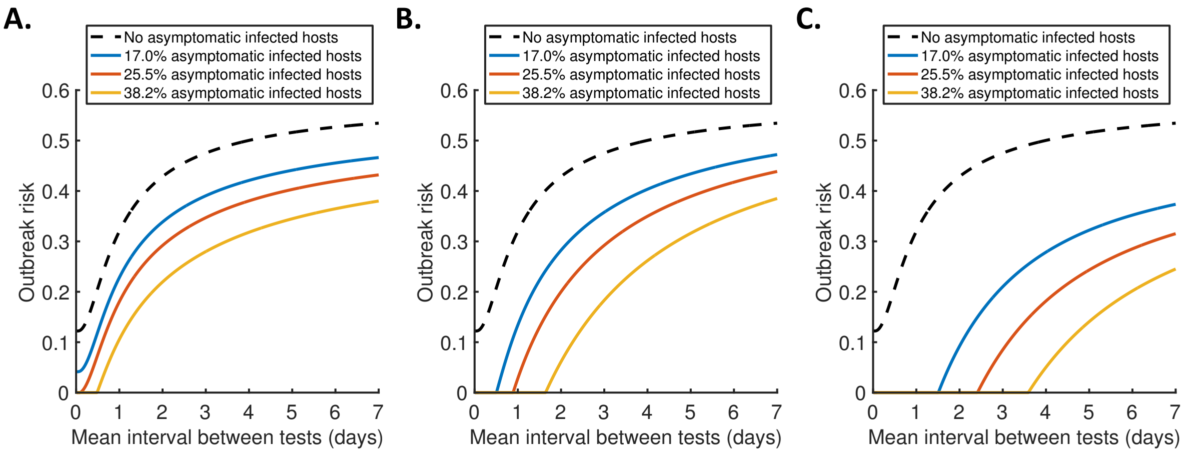

Fig. S5: Effect of the proportion of asymptomatic infected hosts on the outbreak risk under regular antigen testing. A. The outbreak risk for different values of the mean interval between antigen tests, assuming that the proportion of entirely asymptomatic infected hosts is 0% (black dashed), 17.0% (blue), 25.5% (red; as in **Fig. 5**) or 38.2% (orange) – the latter three values represent the lower 95% confidence interval limit, central estimate, and upper confidence limit obtained in a meta-analysis (18), respectively – and that when regular antigen testing does not take place, an asymptomatic infected individual generates (on average) a factor $x_A = 0.32$ times the average number of transmissions generated by an individual who develops symptoms. **B.** Equivalent panel to **A** with $x_A = 1$. **C.** Equivalent panel to **A** with $x_A = 2.77$.

Fig. S6

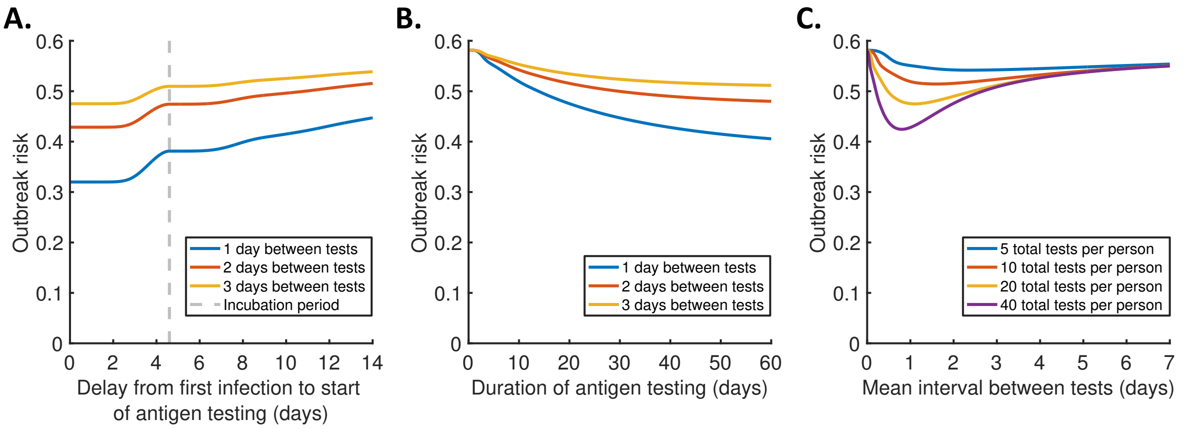

Fig. S6: Effect of delayed and/or time-limited antigen testing. A. The outbreak risk for different delays from the time of the first infection to the introduction of regular antigen testing, assuming an infinite duration of testing, and either 1 (blue), 2 (red) or 3 (orange) days between tests (on average). **B**. The outbreak risk for different durations of antigen testing, assuming a delay of one incubation period (4.6 days) from the first infection to the start of testing (i.e., testing starts following the detection of a symptomatic case), and either 1 (blue), 2 (red) or 3 days (orange) between tests (on average). **C**. The outbreak risk for different values of the mean interval between tests with a total of 5 (blue), 10 (red), 20 (orange) or 40 (purple) tests available to each individual (on average), assuming a delay of one incubation period from the first infection to the start of testing.

SI References

1. Ikeda, H. *et al.* Improving the estimation of the death rate of infected cells from time course data during the acute phase of virus infections: application to acute HIV-1 infection in a humanized mouse model. *Theor Biol Med Model* **11,** 22 (2014).
2. Kim, K. S. *et al.* A quantitative model used to compare within-host SARS-CoV-2, MERS-CoV, and SARS-CoV dynamics provides insights into the pathogenesis and treatment of SARS-CoV-2. *PLoS Biol* **19,** e3001128 (2021).
3. Perelson, A. S. Modelling viral and immune system dynamics. *Nat Rev Immunol* **2,** 28–36 (2002).
4. Hay, J. A. *et al.* Quantifying the impact of immune history and variant on SARS-CoV-2 viral kinetics and infection rebound: a retrospective cohort study. *Elife* **11,** e81849 (2022).
5. Jeong, Y. D. *et al.* Designing isolation guidelines for COVID-19 patients with rapid antigen tests. *Nat Commun* **13,** 4910 (2022).
6. Kuhn, E. & Lavielle, M. Maximum likelihood estimation in nonlinear mixed effects models. *Comput Stat Data Anal* **49,** 1020–1038 (2005).
7. Lixoft. Monolix documentation. at <https://monolix.lixoft.com/>
8. Jeong, Y. D. *et al.* Safely return to schools and offices: early and frequent screening with high sensitivity antigen tests effectively identifies COVID-19 patients. *medRxiv* 2021.10.08.21264782 (2021). doi:10.1101/2021.10.08.21264782
9. Larremore, D. B., Toomre, D. & Parker, R. Modeling the effectiveness of olfactory testing to limit SARS-CoV-2 transmission. *Nat Commun* **12,** 3664 (2021).
10. Larremore, D. B. *et al.* Test sensitivity is secondary to frequency and turnaround time for COVID-19 screening. *Sci Adv* **7,** eabd5393 (2021).
11. Ke, R., Zitzmann, C., Ho, D. D., Ribeiro, R. M. & Perelson, A. S. In vivo kinetics of SARS-CoV-2 infection and its relationship with a person's infectiousness. *Proc Natl Acad Sci U S A* **118,** e2111477118 (2021).
12. Goyal, A., Reeves, D. B., Fabian Cardozo-Ojeda, E., Schiffer, J. T. & Mayer, B. T. Viral load and contact heterogeneity predict SARS-CoV-2 transmission and super-spreading events. *Elife* **10,** e63537 (2021).
13. Heitzman-Breen, N. & Ciupe, S. M. Modeling within-host and aerosol dynamics of SARS-CoV-2: The relationship with infectiousness. *PLoS Comput Biol* **18,** e1009997 (2022).
14. van den Driessche, P. Reproduction numbers of infectious disease models. *Infect Dis Model* **2,** 288–303 (2017).
15. Yates, A., Antia, R. & Regoes, R. R. How do pathogen evolution and host heterogeneity interact in disease emergence? *Proc R Soc B* **273,** 3075–3083 (2006).
16. Norris, J. R. *Markov Chains. Cambridge Series in Statistical and Probabilistic Mathematics* (Cambridge University Press, 1997).
17. Keeling, M. J. & Rohani, P. *Modeling Infectious Diseases in Humans and Animals*. (Princeton University Press, 2008). doi:10.1016/s1473-3099(08)70147-6
18. Yu, W. *et al.* Proportion of asymptomatic infection and nonsevere disease caused by SARS-CoV-2 Omicron variant: A systematic review and analysis. *J Med Virol* **94,** 5790–5801 (2022).
19. Buitrago-Garcia, D. *et al.* Occurrence and transmission potential of asymptomatic and presymptomatic SARS-CoV-2 infections: Update of a living systematic review and meta-analysis. *PLoS Med* **19,** e1003987 (2022).

20. Boucau, J. *et al.* Duration of shedding of culturable virus in SARS-CoV-2 omicron (BA.1) infection. *N Engl J Med* **387,** 275–277 (2022).
21. Hart, W. S. *et al.* Generation time of the alpha and delta SARS-CoV-2 variants: an epidemiological analysis. *Lancet Infect Dis* **22,** 603–610 (2022).
